# Effectively Identifying eQTLs from Multiple Tissues by Combining Mixed Model and Meta-analytic Approaches

**DOI:** 10.1371/journal.pgen.1003491

**Published:** 2013-06-13

**Authors:** Jae Hoon Sul, Buhm Han, Chun Ye, Ted Choi, Eleazar Eskin

**Affiliations:** 1 Computer Science Department, University of California, Los Angeles, California, United States of America; 2 Division of Genetics, Brigham & Women's Hospital, Harvard Medical School, Boston, Massachusetts, United States of America; 3 Broad Institute of MIT and Harvard, Cambridge, Massachusetts, United States of America; 4 Predictive Biology, Inc., San Diego, California, United States of America; 5 Department of Human Genetics, University of California, Los Angeles, California, United States of America; University of California San Diego and The Scripps Research Institute, United States of America

## Abstract

Gene expression data, in conjunction with information on genetic variants, have enabled studies to identify expression quantitative trait loci (eQTLs) or polymorphic locations in the genome that are associated with expression levels. Moreover, recent technological developments and cost decreases have further enabled studies to collect expression data in multiple tissues. One advantage of multiple tissue datasets is that studies can combine results from different tissues to identify eQTLs more accurately than examining each tissue separately. The idea of aggregating results of multiple tissues is closely related to the idea of meta-analysis which aggregates results of multiple genome-wide association studies to improve the power to detect associations. In principle, meta-analysis methods can be used to combine results from multiple tissues. However, eQTLs may have effects in only a single tissue, in all tissues, or in a subset of tissues with possibly different effect sizes. This heterogeneity in terms of effects across multiple tissues presents a key challenge to detect eQTLs. In this paper, we develop a framework that leverages two popular meta-analysis methods that address effect size heterogeneity to detect eQTLs across multiple tissues. We show by using simulations and multiple tissue data from mouse that our approach detects many eQTLs undetected by traditional eQTL methods. Additionally, our method provides an interpretation framework that accurately predicts whether an eQTL has an effect in a particular tissue.

## Author Summary

The combination of gene expression and genetic variation data has enabled the identification of genetic variants that affect gene expression levels. It has been shown that some variants influence gene expression in only one tissue while others influence gene expression in multiple tissues. However, an analysis of multiple tissue data using traditional statistical methods typically fails to identify those variants that affect multiple tissues because each tissue is treated independently and due to low statistical power, the effect in a given tissue may be missed. Building on recent advances in statistical methods for meta-analysis and mixed models, we present a novel method that combines information from multiple tissues to identify genetic variation that affects multiple tissues. We show that our method detects more genetic variation that influences multiple tissues than traditional statistical methods both on simulated and real data.

## Introduction

Advances in genotyping and gene expression technologies have enabled researchers to study associations between genetic variants and gene expression levels. These studies often treat expression levels as quantitative traits and apply statistical tests to identify genomic locations known as expression Quantitative Trait Loci (eQTLs) that segregate the traits. Genome-wide maps of eQTLs for several organisms including budding yeast [1], [2], Arabidopsis [3], mouse [4], [5] and human [6], [7] have been successfully generated. Furthermore, recent technological developments and cost decreases in microarrays allow studies to collect expression data in more than one tissue in human [6], [8], [9] and mouse [4], [5]. A collection of expression data from multiple tissues enables studies to explore the tissue-specific nature of eQTLs as well as their global effects on different types of tissues.

Multiple tissue datasets can potentially allow studies to more effectively identify eQTLs by combining information from multiple tissues. Due to a limited sample size, a standard single tissue eQTL method or “tissue-by-tissue” approach that examines each tissue individually may not detect an eQTL in any one tissue, or it may overestimate the proportion of tissue specific eQTLs [10]. However, if a genetic variant is associated with the expression of a gene in more than one tissue, we can aggregate information from multiple tissues to increase statistical power. This idea is similar to the idea of meta-analysis in genome-wide association studies (GWAS) that combines results of several studies on the same phenotype. In our case, each tissue is considered as a separate “study” in the meta-analysis.

One key difficulty in combining results from multiple tissues is that it is not known in which tissues a genetic variant has an effect. For example, a variant may influence gene expression in all tissues, may have different effects on different tissues, or may have an effect in some tissues but may not have any effect in other tissues. This phenomenon, different effect sizes among tissues, is called heterogeneity. Meta-analysis methods have different assumptions on the distribution of effect sizes, and to better detect eQTLs, studies will perform best if they apply a meta-analysis method whose assumptions are consistent with the actual effect sizes of eQTLs in multiple tissues. For instance, if an eQTL has an effect in all tissues, studies would perform best if they utilize the fixed-effects model (FE) [11]–[13] that assumes no heterogeneity. On the other hand, to effectively detect an eQTL whose effects on gene expression differ across tissues, studies will perform best if they apply the random-effects model (RE) [14]–[18] that considers heterogeneity.

Another challenge in applying meta-analysis to multi-tissue datasets is that studies often collect multiple tissues from the same individuals, which may cause the expression between tissues of the same individual to be correlated. This correlation may cause false positives for standard meta-analysis methods which assume a disjoint set of individuals in each study.

In this paper, we present a novel approach called “Meta-Tissue” that identifies eQTLs from multiple tissues by utilizing meta-analysis. The critical advance of our methodology is that we extend meta-analysis to a mixed model framework. We apply the mixed model to account for the correlation of expression between tissues, and perform meta-analysis to combine results from multiple tissues. Since we do not know in advance the distribution of effect sizes for eQTLs among different tissues, we utilize both the FE and RE models to identify as many eQTLs as possible, and for RE, we use a recently developed random-effects model [18] that achieves higher statistical power than the traditional random-effects model. We first show by simulations that Meta-Tissue is more powerful than the tissue-by-tissue approach in detecting eQTLs when eQTLs have effects in multiple tissues, while controlling for the false positive rate correctly.

We then apply Meta-Tissue to a mouse expression dataset. This dataset is ideal for evaluating methods for discovering eQTLs for several reasons. The data are generated through a cross which limits the genetic diversity in the dataset, and all variants have similar frequencies which eliminate effects of allele frequency on power. In addition, the dataset contains gene expression from many different tissues and different numbers of individuals for the tissues, allowing us to compare results between different scenarios. We analyze four tissues from 50 samples per each tissue and ten tissues from 22 samples. We apply Meta-Tissue to both datasets and demonstrate that Meta-Tissue detects many eQTLs that are undetected by the tissue-by-tissue method.

In addition to accurately detecting eQTLs from multiple tissues, our method can also predict whether an eQTL affects or does not affect expression in a specific tissue. Predicting the existence or absence of an effect is a very difficult problem in meta-analysis, and it is known that making predictions based on p-values is not effective [19]. One of the reasons is that a non-significant p-value is not necessarily evidence of an absence of an effect since the study may be underpowered. Our method instead computes the posterior probability of the presence or absence of an effect for each study building on recent work in interpretation of meta-analysis [19]. Applying the framework to the four and ten tissue datasets, we identify more eQTLs that are predicted to have effects in all tissues compared to the p-value based approach, which are interesting potential candidates with possible global regulatory mechanisms. Meta-Tissue is publicly available at http://genetics.cs.ucla.edu/metatissue/.

## Results

### Meta-Tissue

The main idea of Meta-Tissue is that it combines the effect size estimates from multiple tissues using a “meta-analysis” approach. Meta-analysis techniques are widely applied to combine the results of GWAS studies. In our case, we consider each tissues as a “study.” This has the advantage of increasing the statistical power to detect eQTLs shared across tissues. There are several challenges corresponding to the inherent differences between combining GWAS studies and expression quantitative trait loci studies in multiple tissues. The first challenge is that we expect that there may be differences in effect sizes between tissues. For this reason, we utilize both the random-effects model which allows Meta-Tissue to detect eQTLs when heterogeneity is present, and the fixed-effects model when it is not. A second challenge is that in many multi-tissue eQTL study designs, multiple tissues are collected from the same individuals which induce correlation between measurements of expression levels in different tissues. However, meta-analysis methods assume that studies are independent and may be susceptible to false positives. To overcome this challenge, we utilize the linear mixed model to correct our effect size estimates before performing the meta-analysis.

We assume that multi-tissue eQTL studies collect expression values of 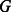 genes from 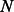 individuals in 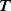 tissues. However, those 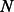 individuals are not necessarily the same for all 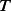 tissues; some individuals may provide a subset of tissues. The studies also collect genotype information of 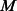 SNPs from the individuals. To determine eQTLs in a specific tissue, or pairs of SNP and gene that are significantly correlated, eQTL studies often use the following linear model.
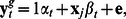
where 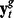 is gene expression 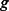 of individuals in tissue 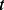, 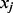 is information on SNP 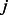, and 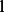 is a vector of ones. 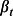 is the effect size of SNP 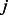 on gene 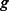 in tissue 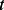, and if it is not zero, we claim the pair of SNP 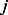 and gene 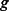 as an eQTL. The Tissue-By-Tissue (TBT) approach computes 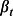 for every tissue (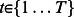), and determines whether at least one 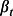 is not zero.

To increase the statistical power to detect eQTLs, Meta-Tissue utilizes meta-analysis that combines 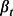 from 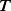 tissues. A naive approach to apply meta-analysis to multi-tissue eQTL datasets is directly using 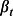 computed from the linear model for TBT. This approach, however, violates the main assumption of meta-analysis that 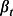 is independent for 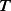 tissues. Because multiple tissues are often collected from the same individuals, there exists correlation between gene expression values across different tissues, and this leads to correlated 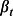.

**Figure 1:**
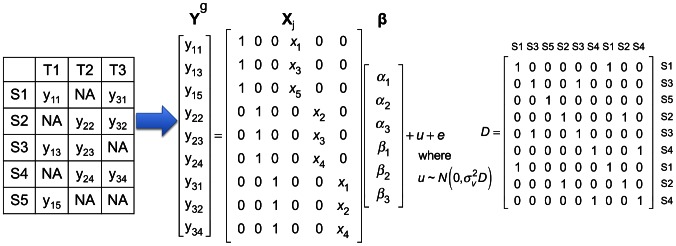
A simple example showing how gene expression and SNP in multi-tissue eQTL studies are encoded in the mixed model of Meta-Tissue. This example has five samples (S1, S2, S3, S4, and S5) in three tissues (T1, T2, and T3). The leftmost table shows which tissues are collected from each sample; 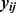 means gene expression of 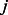
th sample in 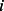
th tissue, and 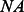 means the tissue is not collected. In this example, each tissue has gene expression measured in three samples. 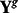 is a vector containing expression of samples in all tissues; there are a total of 9 gene expression values. In the 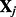 matrix, 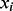 denotes genotype of 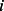
th sample. The 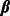 matrix contains three intercepts (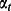) and three 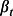 for the three tissues. 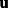 is the random effect of the mixed model, and 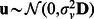. 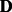 is 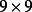 matrix whose entry at 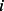
th row and 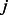
th column is 1 if the 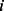
th and 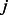
th entries of 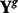 are collected from the same individual, and 0 otherwise.

To apply meta-analysis to correlated 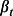, Meta-Tissue uses a linear mixed model to explicitly capture correlation between 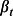:
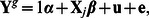
where 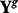 and 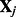 contain gene expression and SNP information in all 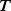 tissues, and Figure 1 shows how they are encoded using a simple example. 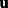 is the random effect of a mixed model due to the fact that multiple tissues are collected from the same individuals. 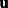 follows the multivariate normal distribution whose covariance matrix (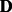 matrix in Figure 1) represents sharing of individuals in multiple tissues. Meta-Tissue applies the generalized least squares to estimate 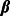 and its covariance or correlation between 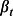. Meta-Tissue “un-correlates” 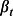 using the covariance it estimated and use the “un-correlated” 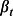 for meta-analysis (see the Materials and Methods section for more details).

There is a fundamental difference between Meta-Tissue and the TBT approach. The statistical test in Meta-Tissue tests whether or not a gene is involved in an eQTL in any of the tissues. In other words, the null hypothesis of Meta-Tissue assumes that no effect is present in any of the tissues for a specific gene. A rejection of this null hypothesis is effectively predicting the presence of an effect in at least one of the tissues. However, the tissue-by-tissue approach tests whether or not an eQTL is present in each tissue. Hence, the null hypothesis of TBT assumes that no effect is present in a specific tissue. This means that Meta-Tissue performs one test per gene and TBT performs one test per gene in each tissue. In our comparisons of Meta-Tissue and TBT, we adjust the significant thresholds so that the overall false positive rate of implicating any tissue of a gene in an eQTL is constant for both methods.

Once we identify a significant association using Meta-Tissue, this means that at least one of the tissues contains an eQTL. In order to identify which subset of the tissues contain an eQTL, we utilize a recently developed meta-analysis interpretation framework which computes an m-value statistic for each tissue [19]. The m-value estimates the posterior probability that an effect is present in a study included in a meta-analysis. Utilizing the m-values, we can predict tissues in which an effect is present.

### Power comparison by simulation

We first simulate gene expression data to compare the power between the traditional Tissue-By-Tissue approach (TBT), Meta-Tissue FE, and Meta-Tissue RE. We create a dataset that has 100 individuals with one SNP and one gene expression level simulating one eQTL. We set the minor allele frequency to 30%. We simulate four tissues and consider four scenarios where a SNP has the same effect in (1) a single tissue, (2) in two tissues, (3) in three tissues, and (4) in all four tissues. The first three scenarios correspond to eQTLs with heterogeneity while eQTLs have no heterogeneity in the last scenario. We check 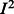 statistics [20] of eQTLs that measure the magnitude of heterogeneity in each scenario and verify that eQTLs have high levels of heterogeneity in the first three scenarios, but very low levels in the last scenario (Figure S1). We assume that each individual provides four tissues, and hence this simulation corresponds to a repeated measures design. We use the mixed model discussed in the Materials and Methods section to generate the gene expression levels of individuals while taking into account the repeated measures design. We generate 1,000 datasets (each a potential eQTL) and the power is estimated as a proportion of eQTLs detected at a significance threshold of 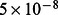 for meta-analysis methods. We choose this threshold because the number of tests we perform in mouse datasets is on the order of one million (135 SNPs
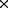
10,588 genes). The significance threshold adjusted for one million tests as in typical GWAS is 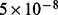. For TBT, we apply a significance threshold of 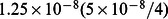 such that the overall false positive rate of TBT is the same as that for Meta-Tissue as discussed in the previous section.

To apply the proposed methods to the simulations, we use the following approach. For TBT, we perform a standard *F*-test using a linear model to obtain a p-value for each pair of a SNP and a gene expression level in each tissue (see Materials and Methods). The tissue-by-tissue approach declares a SNP-gene expression pair as an eQTL if the p-value for the association statistic is below the threshold for any one of the tissues. For Meta-Tissue, we first perform generalized least squares (GLS) to correct for the fact that individuals are shared among tissues. Meta-Tissue then combines information from multiple tissues to obtain either fixed effect or random effect meta-analysis p-values as described in the Materials and Methods section. A SNP-expression pair is considered as an eQTL if its meta-analysis p-value is below the significance threshold. As a separate simulation, we verify that both of our implementations (Meta-Tissue FE and RE) control the false positive rates (Text S1). This simulation also shows that utilizing the mixed model is critical for controlling false positives when expression levels from multiple tissues are collected from the same individual.

**Figure 2:**
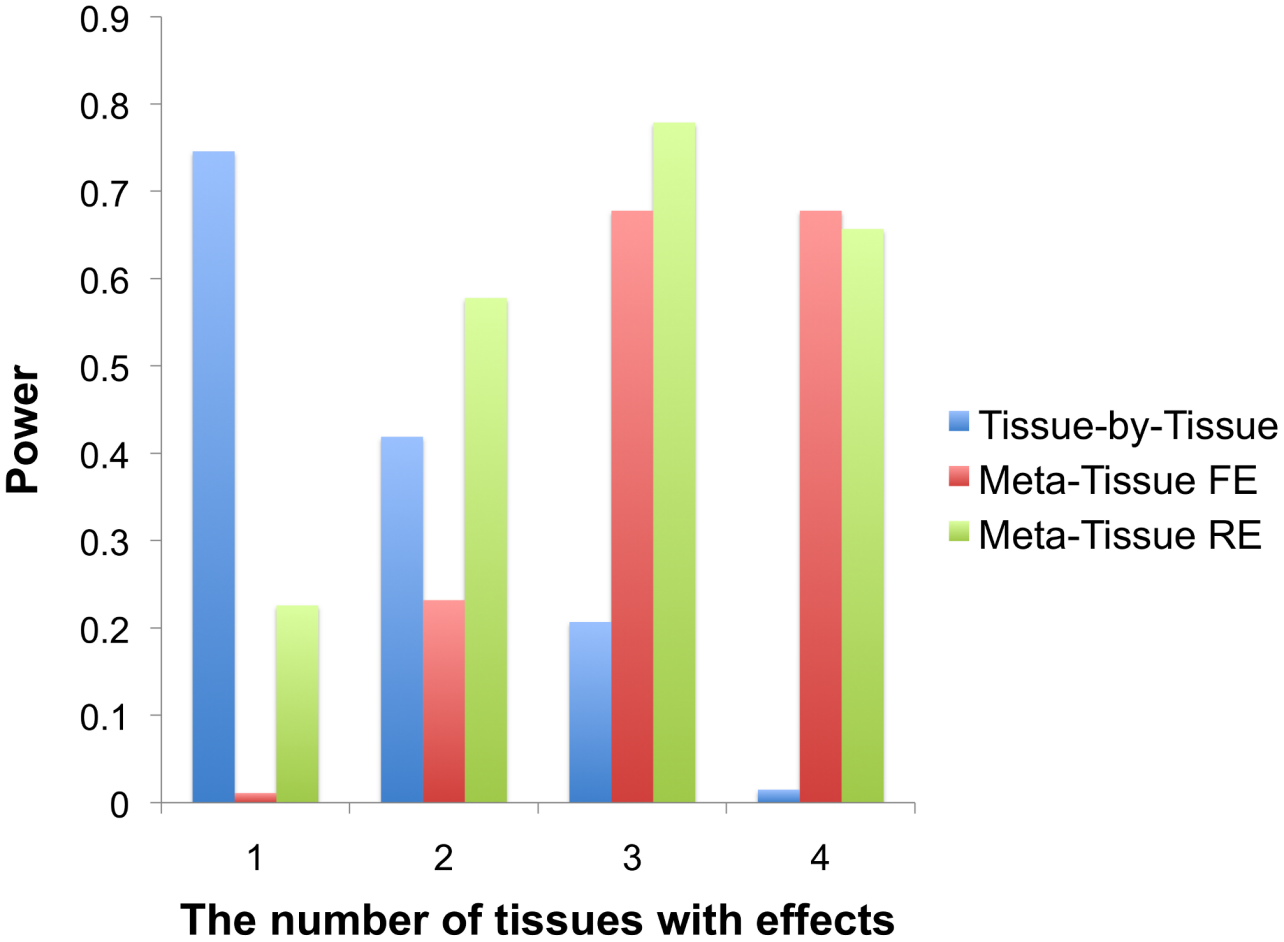
Power comparison between the tissue-by-tissue approach, Meta-Tissue fixed effects model (FE), and Meta-Tissue random effects model (RE) using simulated data. X-axis indicates the number of tissues having effects out of four tissues, and Y-axis is the power.

Figure 2 shows that Meta-Tissue methods are more powerful than TBT when effects exist in multiple tissues; Meta-Tissue RE is the most powerful when an eQTL has effects in two or three tissues, and Meta-Tissue FE outperforms TBT and Meta-Tissue RE when the effects exist in all tissues. The TBT approach has higher power than Meta-Tissue methods when the effects exist in a single tissue. These results show that TBT is an ideal approach to detect an eQTL that is specific to a certain tissue while Meta-Tissue approaches are ideal for detecting an eQTL that has effects in more than one tissue. As the number of tissues with effects increases, the power of Meta-Tissue methods increases while that of TBT decreases. These results suggest an integrated approach in eQTL studies to apply TBT for detecting tissue-specific eQTLs and Meta-Tissue methods for detecting eQTLs shared between tissues.

### Simulation of heterogeneity in multiple tissues using mouse data

To verify the results of the previous power simulation in real multiple tissue data, we simulate heterogeneity using a liver tissue expression from mouse. This dataset contains 108 samples, 135 SNPs and 10,588 probe expression levels. We detect 389 eQTLs in this single tissue dataset using the standard linear model with a p-value threshold of 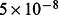, which corresponds to the false discovery rate (FDR) of 0.017% level. We consider these detected associations as the gold standard for measuring accuracy of methods in this simulation. We then split the 108 samples into three groups of 36 samples to simulate three tissues, and this means that eQTLs have effects in all three tissues. In our simulations, we expect to find fewer eQTLs because each of our “tissues” only has 36 samples compared to the original 108 samples. We then consider three scenarios similar to scenarios in the previous power simulation; (1) eQTLs have effects only in the first tissue by permuting expression of the second and third tissues, (2) eQTLs have effects only in the first and second tissues by permuting expression of the third tissue, and (3) eQTLs have effects in all three tissues without any permutation. Permuting the expression of a specific tissue removes effects of eQTLs from the tissue, and hence allows simulation of heterogeneity. We apply Meta-Tissue FE, Meta-Tissue RE, and TBT to this multiple tissue dataset and measure how many eQTLs out of the original 389 eQTLs each method can recover using the same threshold (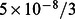 for TBT). Because the number of eQTLs methods recover can change depending on how we split the 108 samples, we perform ten iterations of the experiment where we divide individuals differently in each iteration, and average the results.

**Figure 3:**
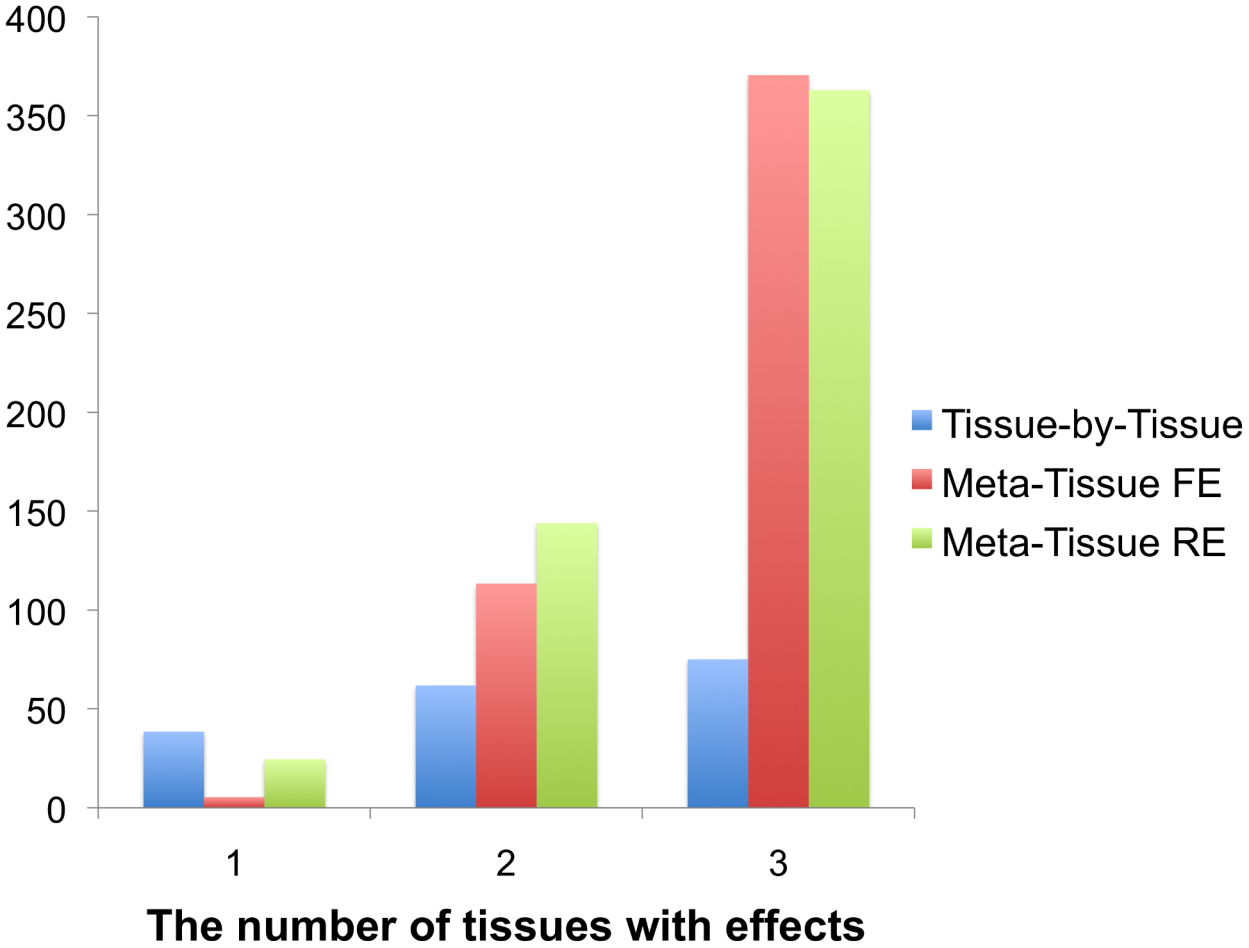
The average number of eQTLs that the tissue-by-tissue approach, Meta-Tissue FE, and Meta-Tissue RE recover from three tissues generated from the liver tissue. The liver tissue has 108 samples from which we simulate three tissues of 36 samples. X-axis indicates the number of tissues having effects out of three tissues. The original liver tissue has 389 eQTLs.

The result of this simulation shows that Meta-Tissue methods recover the most eQTLs when eQTLs have effects in more than one tissue (Figure 3). When effects exist in two out of three tissues, Meta-Tissue RE recovers the most eQTLs; it recovers 144 eQTLs out of the 389 eQTLs on average, and this is 27% and 133% more than the number of eQTLs Meta-Tissue FE and TBT recover, respectively. When eQTLs have effects in all tissues, Meta-Tissue FE recovers the most eQTLs, and when effects exist in a single tissue, TBT does. This result is consistent with the previous power simulation in which Meta-Tissue methods were more powerful than TBT when eQTLs have effects in multiple tissues.

### Detecting eQTLs in multiple tissue mouse data

We apply Meta-Tissue to detect eQTLs in multiple tissues from mouse. Our data consists of two sets; one with four tissues (cortex, heart, liver, spleen), and the other with ten tissues (bone marrow, hippocampus, kidney, pancreas, stomach, white fat, and the four tissues). The four tissue dataset has 50 samples per each tissue while the ten tissue dataset has 22 samples per tissue. In both datasets, not all individuals provided all different types of tissues; on average, 34% of individuals are shared between two tissues in the four tissue dataset while 11% of individuals are shared in the ten tissues dataset. The number of SNPs (135 SNPs) and the number of probes (10,588) are the same as those of the liver tissue.

**Figure 4:**
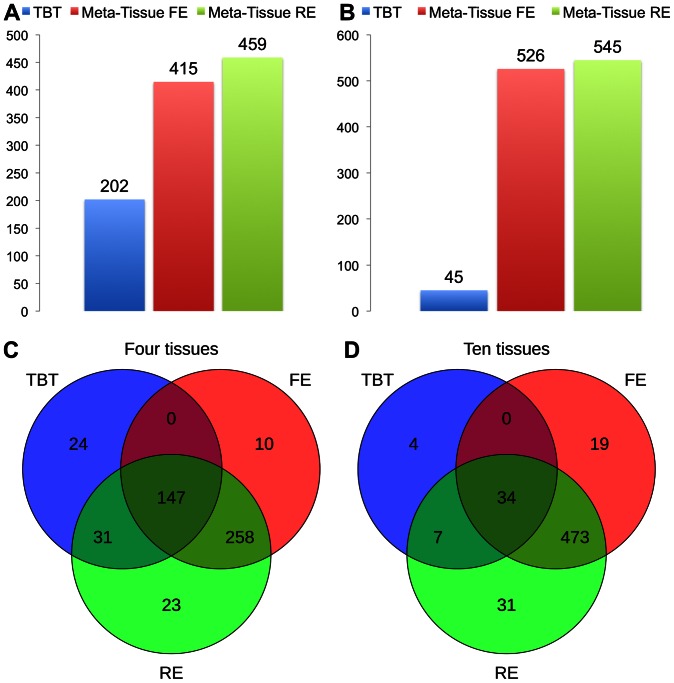
The number of eQTLs detected by the tissue-by-tissue approach (TBT), Meta-Tissue FE, and Meta-Tissue RE in A) four and B) ten tissues of mouse, and the overlap of eQTLs detected by the three methods in C) four and D) ten tissues. The datasets consist of the gene expression levels from 50 individuals (four tissues) and 22 individuals (ten tissues). We apply a p-value threshold of 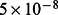 for Meta-Tissue and a threshold of 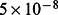/the number of tissues for tissue-by-tissue. The Venn diagrams (C and D) show the number of eQTLs detected by either TBT, FE, or RE, by TBT and either of FE and RE, by FE and RE, and by all three methods.

**Figure 5:**
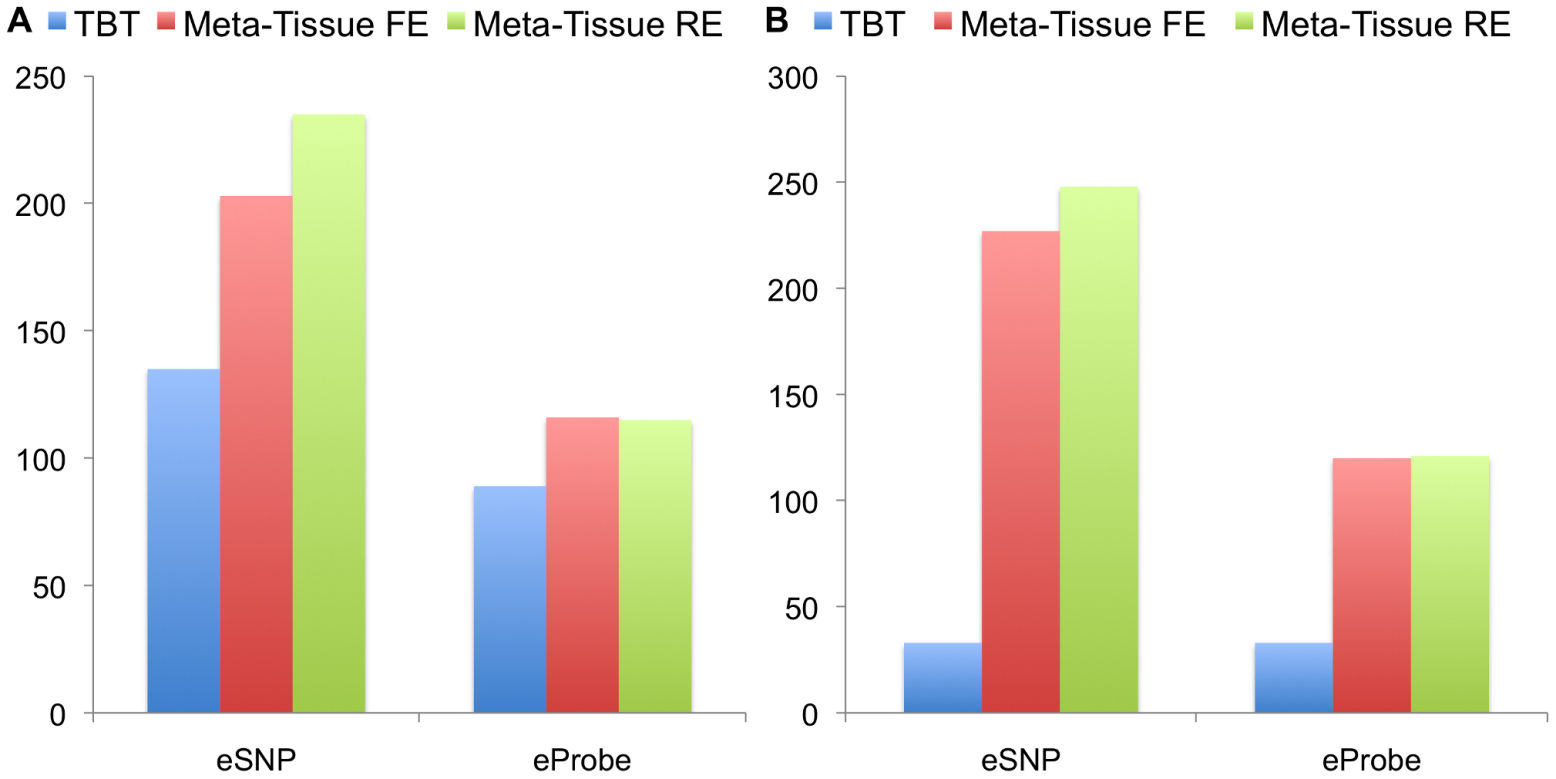
The number of eSNPs and eProbes detected by the tissue-by-tissue (TBT) approach, Meta-Tissue FE, and Meta-Tissue RE in A) four tissues and B) ten tissues of mouse. We apply a p-value threshold of 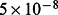 for Meta-Tissue and a threshold of 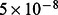/the number of tissues for TBT.

Figures 4A (four tissues) and 4B (ten tissues) show the number of eQTLs detected by Meta-Tissue RE, Meta-Tissue FE, and TBT using a threshold of 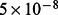 (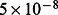/the number of tissues for TBT). The number substantially increases by using Meta-Tissue RE or FE, showing up to two fold and twelve fold increases compared to TBT in the four and ten tissue datasets, respectively. These results indicate that methods that combine results of multiple tissues outperform a method that uses results of each tissue separately as all meta-analysis methods detect more eQTLs than TBT. Moreover, these results suggest a possibility that there exist a considerable number of eQTLs with different effect sizes across tissues as Meta-Tissue RE consistently identifies more eQTLs than Meta-Tissue FE. In addition to the number of eQTLs (SNP-expression pairs), we also analyze the number of eSNPs (unique SNPs influencing gene expression) and eProbes (unique probes for gene expression). Similar to the results of the number of eQTLs, Meta-Tissue detects more eSNPs and eProbes than TBT (Figure 5).

Another important implication comes from comparing the two datasets. TBT finds substantially fewer number of eQTLs in the ten tissue dataset than in the four tissue dataset. This is possibly because the sample size of each tissue is decreased from 50 to 22. On the other hands, the meta-analytic methods find more eQTLs. One possible reason is that the total sample size is slightly increased from 200 to 220. Therefore, the results demonstrate that by using information from multiple tissues and leveraging meta-analysis methods, we may be able to detect eQTLs even if the sample size for each tissue is small.

In addition to the number of eQTLs that different methods detect, we also analyze the overlap of eQTLs using Venn diagrams (Figures 4C and 4D). The Venn diagrams show the number of eQTLs detected only by each of the three methods, by both TBT and each of Meta-Tissue methods, by both Meta-Tissue methods, and by all three methods. In the four tissue dataset, the three methods detect 493 unique eQTLs overall, and a majority of eQTLs (95.1% of total eQTL) are detected by either of Meta-Tissue methods. There are, however, 24 eQTLs (4.9% of total eQTLs) that only TBT detects, and they are likely to be tissue-specific eQTLs. In the ten tissue dataset, almost all eQTLs (99.3% of total eQTLs) are detected by Meta-Tissue RE or FE, and there are 4 eQTLs (0.7% of total eQTLs) detected only by TBT, which may be due to the low statistical power due to the limited number of samples.

Instead of the common genome-wide significance threshold (e.g. 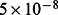) to identify eQTLs, an alternative approach is to use the false discovery rate (FDR) approach, and we use the QVALUE package in R [21] to compute a q-value for each SNP-expression pair. We consider only *cis*-eQTLs for the FDR approach; we consider an eQTL as *cis* if a SNP is on the same chromosome as the probe for gene expression. While typical eQTL studies consider 1 Mb as a distance between a SNP and a probe for *cis*-eQTLs, we consider a much longer distance due to a small number of genotyped SNPs (135 SNPs). Figures S2A and S2B show the number of eQTLs detected by Meta-Tissue methods and TBT using FDR of 0.05 level in four and ten tissues, respectively, and Figures S2C and S2D are Venn digrams showing the overlap of eQTLs. The results using the FDR approach are consistent with those using the common genome-wide significance threshold; Meta-Tissue RE detects most eQTLs among the three methods, and a majority of eQTLs (86% and 93% of total eQTLs for four and ten tissues) are detected either by Meta-Tissue RE or FE.

### Measuring heterogeneity in mouse data

The number of eQTLs detected only by TBT or by RE in Figures 4 and S2 indicates that there can be several eQTLs with different effect sizes in different tissues. To measure the magnitude of heterogeneity of eQTLs, we use the Cochran's Q statistic [14] and the 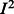 statistic [20]. We make a plot whose x-axis is the 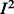 statistic and whose y-axis is the log of p-value of Cochran's Q statistic, and a histogram showing the distribution of 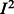 statistics. Figures S3, S4, and S5 show the heterogeneity of eQTLs detected by TBT, FE, and RE, respectively, in the four tissues of mouse data. These plots show that the eQTLs detected by RE show higher level of heterogeneity than the eQTLs detected by FE, as expected. Given the p-value threshold of 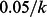 where 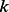 is the number of eQTLs detected, 65, 17, and 53 eQTLs show statistically significant heterogeneity in TBT, Meta-Tissue FE, and Meta-Tissue RE, respectively, using the p-value of Cochran's Q statistic.

### Predicting the presence of effects in multiple tissue data

Our Meta-Tissue approach not only detects more eQTLs from multiple tissues but also provides an interpretation framework that predicts whether an eQTL has effects in a specific tissue. Meta-Tissue computes a statistic called m-value [19], and it is the posterior probability that an effect exists in a specific tissue. If the m-value is greater than a threshold 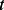, we predict that an effect exists, and if it is less than 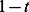, we predict that an effect does not exist. Another approach to predict an effect is to use a p-value. In this approach, an effect exists if a p-value is less than a significance threshold and does not exist otherwise.

We first apply this prediction framework to the 3-way split liver tissue dataset that we previously generated. Recall that the liver tissue has 389 eQTLs, and we simulated three tissues from it and three scenarios in which we varied heterogeneity of eQTLs. For this simulation, we consider only the scenario where eQTLs have effects in the first two tissues out of three since this corresponds to heterogeneity in which the number of eQTLs that TBT and Meta-Tissue recover is relatively large. We measure how accurately Meta-Tissue and the p-value approach predict the presence and absence of effects of the 389 eQTLs in the three tissues. More specifically, Meta-Tissue makes a correct prediction if m-values are greater than 0.9 in the first two tissues and the m-value is less than 0.1 in the third tissue (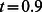). We consider an m-value prediction to be ambiguous if any of the three tissues has the m-value between 0.1 and 0.9. If the prediction is not either correct or ambiguous, it is considered as an incorrect prediction. For the p-value approach, p-values of the first two tissues need to be less than the significance threshold (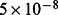/3) and p-value of the third tissue needs to be greater than the threshold for a correct prediction. Otherwise, the prediction is an incorrect prediction since the p-value approach does not have the notion of the ambiguous prediction. In the original 3-way split liver tissue experiment, we had ten simulations which differed in how the individuals were divided. Over the ten simulations, Meta-Tissue and TBT recovered 146 eQTLs out of total 389 eQTLs on average (Figure 3). Since we use m-values for the interpretation purpose (not for detecting eQTLs), we apply m-values to only those 146 eQTLs. We also predict effects of the 146 eQTLs using the p-value approach.

Meta-Tissue makes the correct prediction for 35% (51/146) of the eQTLs and predicts the ambiguous prediction for 56% (82/146). The p-value approach only makes the correct prediction for 11% (16/146) of the eQTLs. The number of correct predictions of Meta-Tissue is more than three times greater. In addition, given the advantage of the fact that Meta-Tissue can make ambiguous predictions, the number of incorrect predictions for Meta-Tissue (13/146) is ten times fewer than that for the p-value approach (130/146). The results demonstrate that by combining the meta-analysis method and the interpretation framework, we may predict effects of eQTLs more accurately than the approach utilizing p-values.

We then apply our interpretation framework to the four and ten multiple tissue datasets from mouse to predict effects of eQTLs that were discovered using Meta-Tissue and TBT (493 and 568 eQTLs in four and ten tissue datasets, respectively). We calculate the m-value for each eQTL per each tissue and make a prediction that the eQTL affects expression in that tissue if the m-value is greater than 0.9. We also compare our approach to the p-value approach as in the previous simulation using the same threshold (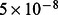/the number of tissues).

First, we apply the two approaches to the four tissue dataset, and Table 1 lists the number of eQTLs predicted to have effects across various combinations of tissues (e.g. eQTLs affecting expression in heart/liver, heart/cortex, heart/liver/cortex). The results show that Meta-Tissue consistently categorizes more eQTLs having effects in multiple tissues than the p-value approach. Among those eQTLs, ones that influence expression levels in all tissues are particularly interesting because they may provide insights into the global regulatory mechanisms of eQTLs. Meta-Tissue predicts 283 such eQTLs while the p-value approach predicts 15 eQTLs. The small number of predictions in p-value approach is expected because even if the effect exists in all 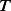 tissues, given power 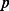 of tissue-by-tissue approach, we can predict the global effect only with probability 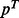.

**Table 1:** The number of eQTLs predicted to have effects by Meta-Tissue and the p-value approach across various combinations of the four tissues.

Tissues	Meta-Tissue	p-values
Cortex/Heart	7	6
Cortex/Liver	1	2
Cortex/Spleen	4	2
Heart/Liver	7	3
Heart/Spleen	7	4
Liver/Spleen	10	2
Cortex/Heart/Liver	28	7
Cortex/Heart/Spleen	49	1
Cortex/Liver/Spleen	17	0
Heart/Liver/Spleen	24	2
All four tissues	283	15

Meta-Tissue uses m-value statistics to predict effects; if m-value is greater than 0.9, the effect exists. The p-value approach uses p-values to make predictions; the effect exists if p-value is less than the significance threshold (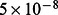/the number of tissues).

We next predict effects of eQTLs in the ten tissue dataset, and for this dataset, we would expect to detect a fewer number of eQTLs having effects across all tissues since it becomes less likely that all p-values or m-values pass the threshold as we try to detect effects in more tissues. Table 2 shows the number of eQTLs predicted to affect expression across different numbers of tissues considered (e.g. eQTLs having effects across any two tissues, any three tissues). Similar to the results of the four tissue dataset, Meta-Tissue predicts more eQTLs with effects in several tissues than the p-value approach. Unlike the four tissues, we detect a fewer number of eQTLs having effects in all ten tissues; 134 and zero such eQTLs by Meta-Tissue and the p-value approach, respectively. The results indicate the intrinsic difficulty in detecting eQTLs influencing expression across many different tissues.

**Table 2:** The number of eQTLs predicted to have effects by Meta-Tissue and the p-value approach across different numbers of tissues considered in the ten tissue dataset (eQTLs having effects across any two tissues, any three tissues, etc.).

	Meta-Tissue	p-values
2 tissues	12	10
3 tissues	7	0
4 tissues	20	4
5 tissues	33	0
6 tissues	36	1
7 tissues	88	0
8 tissues	99	0
9 tissues	124	0
10 tissues	134	0

## Discussion

We presented a statistically powerful approach to detect eQTLs from multiple tissues. Our approach, Meta-Tissue, takes advantage of two meta-analysis methods that differ in their assumptions on effects of eQTLs in different tissues. The first method assumes that effects exist in all tissues with the same magnitude, and this assumption allows us to detect eQTLs shared across all tissues. The second method assumes that effect sizes of variants are different among studies. By assuming the heterogeneity, we may be able to accurately describe the nature of eQTLs whose patterns of genetic regulation differ across tissues. Meta-analysis methods, however, assume that studies are independent, and this assumption is unlikely to be true in multi-tissue dataset since studies collect multiple tissues from the same individuals. This may cause correlation in expression between tissues, and to correct for the correlation, we utilized a mixed model that enables the meta-analysis method to achieve correct false positive rates.

To measure the performance of Meta-Tissue, we first showed by simulations that our methods are generally more powerful than a naive approach that looks at results of each tissue individually. Next, by using data from mouse liver tissue, we simulated the heterogeneity in effect sizes across a subset of tissues as well as in all tissues. Meta-Tissue methods were shown to recover more original eQTLs from multiple tissues than the naive tissue-by-tissue approach when effects exist in multiple tissues. We then observed that Meta-Tissue detects many eQTLs that the naive approach does not detect in four and ten tissue datasets from mouse. However, we note that there are a few tissue-specific eQTLs that only the naive approach detects, and hence we recommend that eQTL studies also apply the naive approach in addition to Meta-Tissue.

In addition to detecting more eQTLs, Meta-Tissue can also accurately predict whether an effect exists in a specific tissue. Meta-Tissue calculates the posterior probability that an eQTL has an effect in a certain tissue, and we demonstrated that this probability is more effective in predicting the effect than a p-value is by using the same liver tissue simulation. We then predicted effects of eQTLs that we found in the four and ten tissue datasets and showed our method predicts more eQTLs having effects in multiple tissues than the p-value approach.

Our approach is fundamentally different from previous approaches that also attempt to detect eQTLs from multiple tissues, and to the best of our knowledge, Meta-Tissue is the first method to apply both a mixed model and meta-analysis methods to eQTL mapping. A traditional approach to detect associations from repeated measurements from same individuals such as multiple tissue data is MANOVA. However, MANOVA is not directly applicable to our multiple tissue data because not all samples provided all different types of tissues, and hence our data are not completely “repeated measurements.” Meta-Tissue is more general than MANOVA since Meta-Tissue can be applied to both “repeated measures design” in which individuals are shared across all tissues and to a scenario in which only a subset of individuals are shared. Another advantage of our method is that Meta-Tissue can take into account population structure by adding an additional variance component term in our mixed model. This may be important to multiple tissue datasets in which individuals are sampled from different populations, which may cause inflation of false positives.

Meta-Tissue leverages the recently developed random effects model [18] that achieves higher power than the traditional random effects model [14]–[17]. Han and Eskin showed that the traditional random effects model never achieves higher power than the fixed effects model due to its conservative null hypothesis. We apply the traditional RE to our power simulation (Figure S6), the heterogeneity experiment with the liver tissue (Figure S7), and the four and ten tissue datasets of mouse data (Figure S8), and we observe the same phenomenon; the traditional RE is always less powerful than FE and the recently developed RE.

There are a few other methods that attempt to detect eQTLs from the multiple tissue data such as Sparse Bayesian Multiple Regression and the GFlasso approach proposed by Petretto et al. [22] and Kim et al. [23] However, a key difference between these methods and Meta-Tissue is that they attempt to detect multiple variants (“multi-locus”) associated with multiple traits while our method focuses on an association of a single variant. Another difference and one main advantage of Meta-Tissue is that since it is a meta-analysis method, studies can combine results of many published eQTL analyses without actual data assuming that those analyses are independent; only results of an eQTL analysis such as effect size estimates are needed when the analyses are independent. Meta-Tissue has another advantage that it is simpler and more computationally efficient than other methods that involve computationally challenging algorithms such as Bayesian variable selection and regularized linear regression including Lasso. While we applied Meta-Tissue to the multi-tissue dataset with a small number of genotyped SNPs and samples (135 SNPs and about a total of 200 samples across tissues), our algorithm and software are efficient enough to be applied to larger eQTL studies where there are hundreds of individuals genotyped at hundreds of thousands SNPs.

## Materials and Methods

### Mouse strains

**Figure 6:**
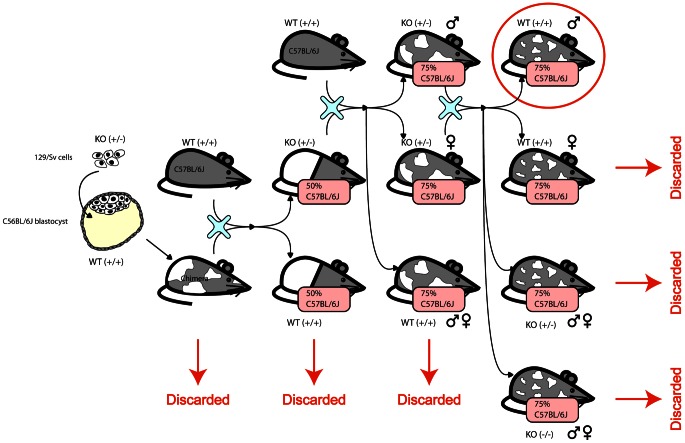
The mice were generated by creating a chimera with heterozygous 129/Sv cells in a C56Bl/6J blastocyst. The chimera was crossed with a wildtype C56Bl/6J to obtain heterozygous KOs and homozygous WTs. The heterozygous KOs were backcrossed to wildtype C56Bl/6J to obtain animals that are 75% C56Bl/6J. The male and female heterozygous KOs are intercrossed and only the resulting wildtype males are used in this study. The complicated structure of the cross is due to the fact that the knockouts were designed to be used subsequently for other studies.

F1N2 mice from a C57BL6/N×129/OlaHsd cross were produced as follows. Male ES cell chimeric founders (E14 ES line [24]) were crossed to C57BL6/N females (Harlan Laboratories). Male agouti offspring were backcrossed to C57BL6/N females, and F1N1 offspring were intercrossed to produce F1N2 animals, Figure 6. All animals were maintained in ventilated microisolator caging (Allentown), fed a standard lab chow diet (Harlan Teklad) and provided water ad libidem. F1N2 animals were group housed with littermates until 9 weeks of age. Mice selected for tissue harvest were singly housed for one additional week, to minimize socialization effects. Only males were used, to avoid estrus related effects on gene expression. While the production crosses segregated various gene targeted alleles, all mice selected for this study carried only wild type genomes and did not carry any engineered genomic alterations such as gene knockouts.

### Gene expression

Animals were sacrificed by cervical dislocation and immediately dissected. A set of thirty tissues were collected from each animal in a prescribed order, beginning with the pancreas. Each tissue was briefly rinsed in PBS and deposited in RNAlater (Ambion), held at room temperature to allow diffusion of RNAlater into the tissue, and then stored at −86C.

Tissue homogenization, total RNA isolation, cDNA production, in vitro transcription and fluorescent labeling were performed as per Affymetrix gene chip recommended protocols. The hybridization mixes were analyzed using Affymetrix U74Av2 expression microarrays, washed and scanned using Affymetrix instrumentation and protocols.

We consider the 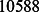 probes for which we have annotations. For each tissue type, we filter out array outliers which show an average correlation of 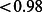 with respect to all other arrays.

The mice were genotyped at 140 SNPs that are polymorphic between 129S1/SvImJ and C57BL/6J from the JAX SNP Genotyping Panel [25]. Information on SNPs is listed in Table S1. We use 135 out of the 140 SNPs that are polymorphic in all tissues for our analysis.

### Normalization and selection of individuals

In our analysis, we consider the gene expression levels of 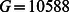 probes collected in 4 tissues (liver, spleen, cortex and heart) over 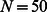 individuals. To be consistent with the different tissue datasets we analyze, we randomly chose 50 individuals from those datasets that have more than 50 individuals. We first used RMA to perform background adjustment on the raw expressions and then quantile normalization to normalize the adjusted expressions. For 10 tissues, we collect the same number of gene expression levels over 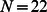 individuals.

### Power simulation framework

Our power simulation assumes that we collect four tissues from 100 individuals, and considers four scenarios where an eQTL has an effect in (1) one tissue, (2) in two tissues, (3) in three tissues, and (4) in all four tissues. To generate the gene expression level of individuals that considers the repeated measurements from the same individuals, we first sample gene expression from the multivariate normal distribution:
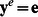
(1)where 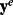 is a vector of size 400 corresponding to gene expression of 100 individuals in 4 tissues, and 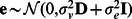 where 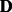 is a 400 by 400 matrix representing correlation between individuals across the tissues. More specifically, 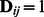 if 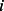 and 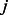 are the same individual between two tissues, and 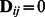 otherwise. 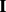 is an identity matrix with size of 400. 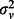 and 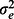 are coefficients of the two variance components, and we use the real mouse dataset to obtain realistic values of the two coefficients. We estimate 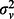 and 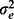 for every pair between a gene expression and a SNP, and find that on average, 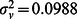 and 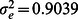. We use these values for our simulation.

After sampling 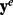, we add a SNP effect to 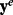 for tissues in which an effect exists using the following equation:
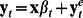
where 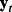 is gene expression of 100 individuals in tissue 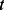 (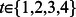), 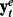 is 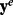 on tissue 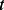 (size of 100), and 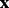 is SNP information of 100 individuals. 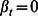 if an eQTL does not have an effect in tissue 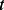, and 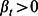 if an eQTL has an effect. Since the goal is to compare the relative power between methods, we vary the effect size (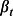) depending on the scenario to avoid too high or too low power. Specifically, we set 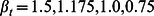 for the scenarios (1), (2), (3), (4), respectively.

### Linear model for tissue-by-tissue approach

We assume an additive linear model to represent the relationship between the expression of one gene and one SNP. We can write that relationship in the following way for an arbitrary gene 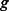 and SNP 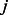 at tissue 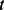:
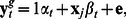
(2)where 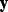 is a size 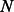 vector denoting gene expression levels of 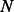 individuals, 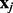 is a size 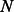 vector denoting SNP, 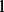 is a vector of ones, and 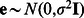. To assess the significance of an association between a SNP and a gene, we perform a standard *F*-test for the null hypothesis 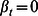 and also obtain an estimate of 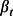 using the lm function in R. In the tissue-by-tissue approach, if any single tissue turns out to be significant (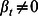), the pair of SNP and gene expression are reported as a significant eQTL. TBT can also find tissues in which an eQTL exists by examining which 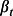 is non-zero.

### Meta-Tissue - linear mixed model

We use a linear mixed model to take into account the fact that eQTL studies collect multiple tissues from the same individuals. This is called a “repeated measures design,” and the mixed model is often used to model the correlation induced by the repeated measurements such as in longitudinal data. Let 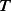 be the number of tissues, and for simplicity, we assume there are 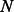 individuals for each tissue, but individuals collected in one tissue do not necessarily completely overlap with those in another tissue; it is possible that some individuals may provide all tissues while others may provide a subset. We also assume that we have SNP information for all individuals. We apply the following linear mixed model to assess the statistical significance between gene expression 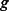 and SNP 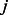:
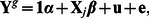
(3)Here is a description of each variable in above equation. Let 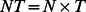.

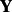
 is an 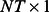 matrix denoting expression levels of 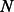 individuals in 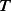 tissues. In other words, the first 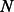 rows are expression of 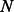 individuals in the first tissue, the next 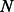 are expression in the second tissue, and so on. Expression values of each tissue are normalized to 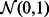.

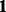
 is an 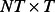 matrix denoting the intercepts for 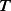 tissues. The first column of 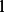 denotes the intercept for the first tissue; the first 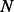 rows are ones, and the next 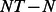 are zeros. In the second column that denotes the intercept for the second tissue, the first 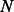 rows are zeros, the next 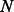 rows are ones, and the next 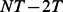 rows are zeros.

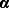
 is a 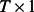 matrix denoting coefficients of intercepts.

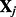
 is an 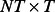 matrix denoting SNP for 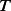 tissues. This is similar to the 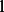 matrix, and we replace ones in the 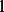 matrix with SNP information. For example, in the first column, the first 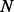 rows are SNP information of 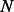 individuals in the first tissue, and the next 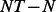 rows are zeros.

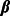
 is a 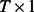 matrix denoting coefficients of SNP effects in 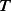 tissues.

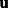
 is the random effect of the mixed model due to the repeated measurements of individuals, and 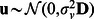 where 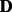 is an 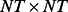 matrix representing how individuals are shared across the tissues (discussed in the Power simulation framework section). 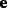 represents random errors and 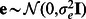 where 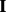 is an identity matrix. To efficiently estimate the two variance components (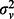 and 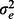), we use the efficient mixed-model association (EMMA) package [26].

To estimate 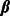 and its covariance, we apply the generalized least squares. Let 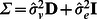. Then, the estimated 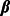 is
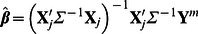
(4)


### Meta-Tissue - meta-analysis

Given the estimate 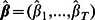, we combine information from multiple tissues by applying meta-analysis to 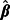. If the effect of eQTL is the same for all tissues, applying fixed effects model (FE) meta-analysis will be a powerful approach. If the effects of eQTL differs by tissues, applying random effects model (RE) meta-analysis will be a powerful approach [18].

#### Fixed effects model

Fixed effects model (FE) is a meta-analysis method that assumes the effect size of a variant is fixed across datasets [12], [13], and its statistic is computed based on the inverse-variance-weighted effect size [27]. Let 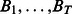 and 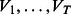 be the estimates of effect-size and the standard error of 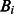, respectively, in 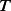 tissues. Let 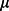 be the unknown true effect size. The null hypothesis of FE is 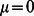; in other words, effect size in all tissues is zero. A statistic of FE (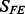) and its distribution under the null hypothesis are
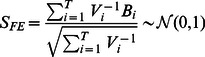
(5)A p-value of 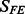 is obtained from the standard normal distribution.

#### Random effects model

Our Meta-Tissue method leverages new random effects model (RE) [18] to detect eQTLs from multiple tissues while taking into account heterogeneity of effect sizes in different tissues. The assumption of the random effects model is that the effect size of a variant is different among datasets and follows a probability distribution with mean 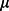 and variance 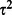. The null hypothesis of the random effects model is equivalent to that of the fixed effects model; that is, 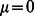. The traditional random effects model, however, assumes a conservative null hypothesis model. The new random effects model corrects this conservative null hypothesis model and outperforms the traditional random effects model. More specifically, a statistic of RE (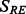) is defined as
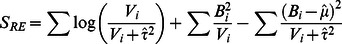
(6)where 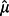 and 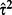 are estimated mean and variance of the effect size, and the maximum likelihood estimates of the two parameters are calculated iteratively as following
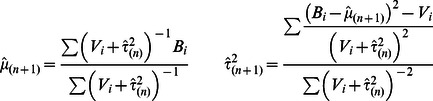
The initial value of 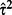 is estimated using approaches in the traditional random effects model [14], [20], [28]. We obtain a p-value of 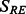 from p-value tables that are constructed from numerous null statistics.

#### Accounting for covariance of effect size estimates

Since we use linear mixed model to account for the fact that multi-tissue eQTL studies often collect multiple tissues from the same individuals, our estimates of effect size, 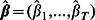 in Equation (4) can become correlated. The covariance structure is estimated using the standard formula of the generalized least squares,
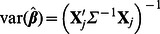
(7)It is important that the meta-analysis methods account for this covariance structure of effect size estimates.

To take into account the covariance structure in meta-analysis, we use an extension [29] of the Lin and Sullivan approach [30]. Given 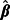 and their covariance 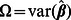, the optimal fixed effects model meta-analysis statistic is
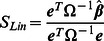
where 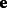 is the vector of ones (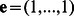). The variance of the statistic is given
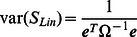
Note that if 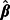 is independent (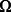 is a diagonal matrix), 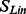 and 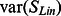 are equivalent to the inverse-variance weighted effect size estimate (the numerator of equation (5)) and its variance.

It can be shown that this approach is equivalent to building a new “un-correlated” variance of 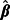,
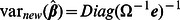
and then giving 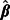 and 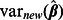 as input to the traditional meta-analysis approaches assuming independent estimates [29]. This “un-correlating” idea allows us flexibility to use the correlated estimates in any meta-analysis framework requiring independent estimates. We use 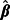 and its “un-correlated” variance for the fixed effects model (which gives equivalent results to the Lin and Sullivan approach [30]), random effects model, heterogeneity estimation (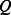 and 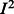), and the m-value estimation [19].

#### Predicting effects of eQTLs in multiple tissues

To predict whether an eQTL has effects in a specific tissue, Meta-Tissue computes a statistic called the “m-value” proposed by Han and Eskin [19] that specifies the posterior probability that an effect exists in a tissue. First, we denote 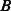 as a vector of 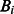; 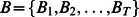. Let 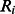 be a random variable whose value is 1 if dataset 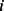 has an effect and 0 otherwise. We also denote 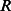 as a vector of 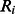, and since each 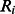 has two values, 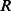 has 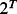 possible values. Let 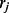 be one of those 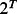 values, and let 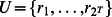 denote a vector of 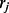. To estimate the m-value 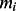, we need to compute the probability, 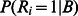, which is the probability of dataset 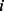 having effects given the observed effect sizes. We can compute this probability using the Bayes' theorem
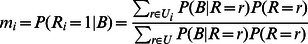
where 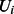 is a set of 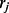 in which 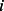
th value is 1. The equation shows that we need to compute 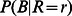 and 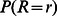 terms for every 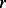 to compute 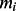. We can compute 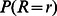 as
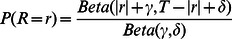
where 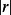 denotes the number of 1's in 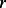 and 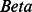 denotes the beta function. 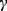 and 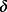 are set to one [19]. The probability of 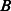 given 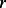, 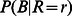, is computed as
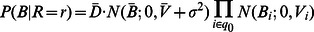
where
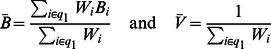


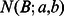
 denotes the probability density function of the normal distribution with mean equal to 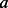 and variance equal to 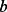, and 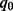 and 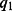 denote the indices of 0 and 1 in 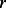, respectively. 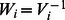 is the inverse variance, and 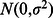 is the prior for the effect size; 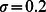 when an effect is small while 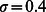 when an effect is large for binary traits [31], [32]. For quantitative traits, there is no general guidelines for the normally distributed priors, so we choose to use the default value 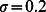. 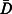 is a scaling factor defined as
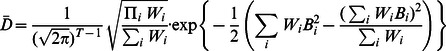
More detailed derivations of 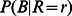 and 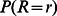 terms are discussed in Han and Eskin [19].

### Practical issues in combining mixed model and meta-analysis

There are subtle issues in our framework combining mixed model and meta-analysis. First, the effect size estimates from linear model or mixed model are typically 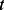-distributed, while most of meta-analysis methods assume normally distributed effect sizes. Second, our approach simultaneously considers all tissues using Equation (3), but the error model is slightly different from the tissue-by-tissue approach in Equation (2). In the tissue-by-tissue approach, the error 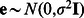 is fit in each tissue separately, while in our new approach, the error is fit in all tissues together, which is often less powerful than the former. We correct for these subtle differences using simple heuristics (See Text S2).

### Ethics statement

This study was performed in strict accordance with the recommendations in the Guide for the Care and Use of Laboratory Animals of the National Institutes of Health.

## Supporting Information

Figure S1 Histograms showing the distribution of I2 statistics in the power simulation. There are four scenarios in the power simulation where an eQTL has an effect 1) in one tissue, 2) in two tissues, 3) in three tissues, and 4) in all four tissues. There are 1,000 eQTLs in each scenario, and the histograms show the distribution of I2 statistics of the 1,000 eQTLs.
(TIF)

Figure S2 The number of eQTLs detected by the tissue-by-tissue approach (TBT), Meta-Tissue FE, and Meta-Tissue RE in A) four and B) ten tissues of mouse using FDR of 5%, and the overlap of eQTLs detected by the three methods in C) four and D) ten tissues. We consider only cis-eQTLs for the FDR approach, and a pair of SNP-probe for gene expression are considered cis if a SNP and a probe are on the same chromosome.
(TIF)

Figure S3 A plot showing heterogeneity of eQTLs detected by the tissue-by-tissue approach. X-axis of the top plot indicates I2 statistic and Y-axis indicates log of p-value of Cochrans Q statistic. The vertical dashed line is drawn at I2 = 50%, and the horizontal dash line is drawn at p-value = 0.05/the number of eQTLs detected. The bottom histogram shows the distribution of I2 statistic.
(TIF)

Figure S4 A plot showing heterogeneity of eQTLs detected by Meta-Tissue FE. X-axis of the top plot indicates I2 statistic and Y-axis indicates log of p-value of Cochrans Q statistic. The vertical dashed line is drawn at I2 = 50%, and the horizontal dash line is drawn at p-value = 0.05/the number of eQTLs detected. The bottom histogram shows the distribution of I2 statistic.
(TIF)

Figure S5 A plot showing heterogeneity of eQTLs detected by Meta-Tissue RE. X-axis of the top plot indicates I2 statistic and Y-axis indicates log of p-value of Cochrans Q statistic. The vertical dashed line is drawn at I2 = 50%, and the horizontal dash line is drawn at p-value = 0.05/the number of eQTLs detected. The bottom histogram shows the distribution of I2 statistic.
(TIF)

Figure S6 Power comparison between the tissue-by-tissue approach, Meta-Tissue fixed effects model (FE), Meta-Tissue random effects model (RE), and Meta-Tissue traditional random effects model using simulated data. X-axis indicates the number of tissues having effects out of four tissues, and Y-axis is the power.
(TIF)

Figure S7 The average number of eQTLs that the tissue-by-tissue approach, Meta-Tissue FE, Meta-Tissue RE, and Meta-Tissue traditional RE recover from three tissues generated from the liver tissue. Effects of eQTLs exist in only two tissues. The original liver tissue has 389 eQTLs.
(TIF)

Figure S8 The number of eQTLs detected by the tissue-by-tissue approach, Meta-Tissue FE, Meta-Tissue RE, and Meta-Tissue traditional RE in A) four tissues and in B) ten tissues of mouse.
(TIF)

Table S1 Information on genotyped SNPs (chromosome, ID, and position).
(XLS)

Text S1 False positive rates of Meta-Tissue.
(PDF)

Text S2 Practical issues in combining mixed model and meta-analysis.
(PDF)
