# Supplementary figures and images for: Effectively Identifying eQTLs from Multiple Tissues by Combining Mixed Model and Meta-analytic Approaches
Source: PLoS Genet. 2013 Jun 13;9(6):e1003491. doi: 10.1371/journal.pgen.1003491 (PMC3681686; doi:10.1371/journal.pgen.1003491)

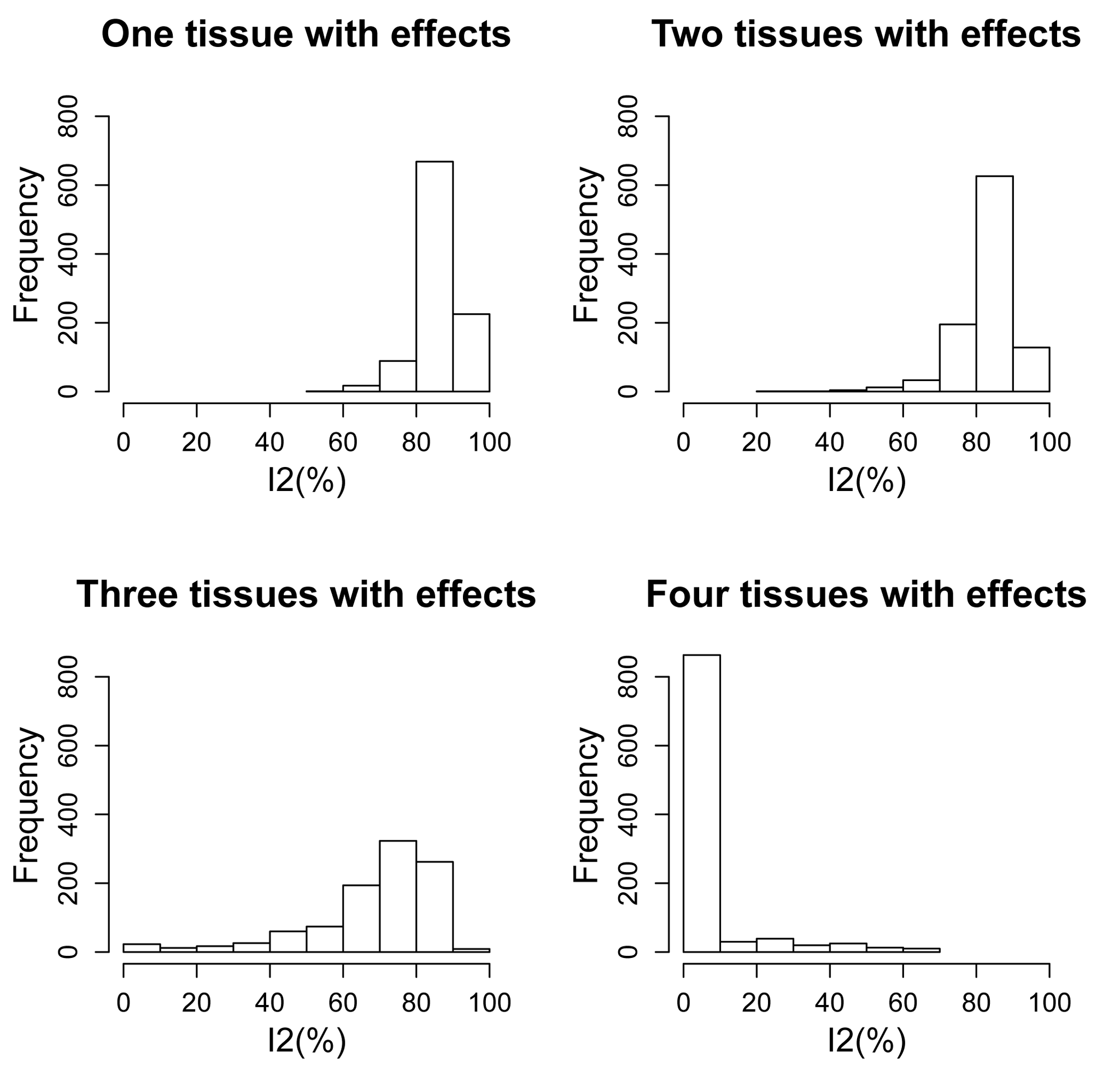

Supplement: Figure S1 — Histograms showing the distribution of I2 statistics in the power simulation. There are four scenarios in the power simulation where an eQTL has an effect 1) in one tissue, 2) in two tissues, 3) in three tissues, and 4) in all four tissues. There are 1,000 eQTLs in each scenario, and the histograms show the distribution of I2 statistics of the 1,000 eQTLs. (TIF) [file pgen.1003491.s001.tif]

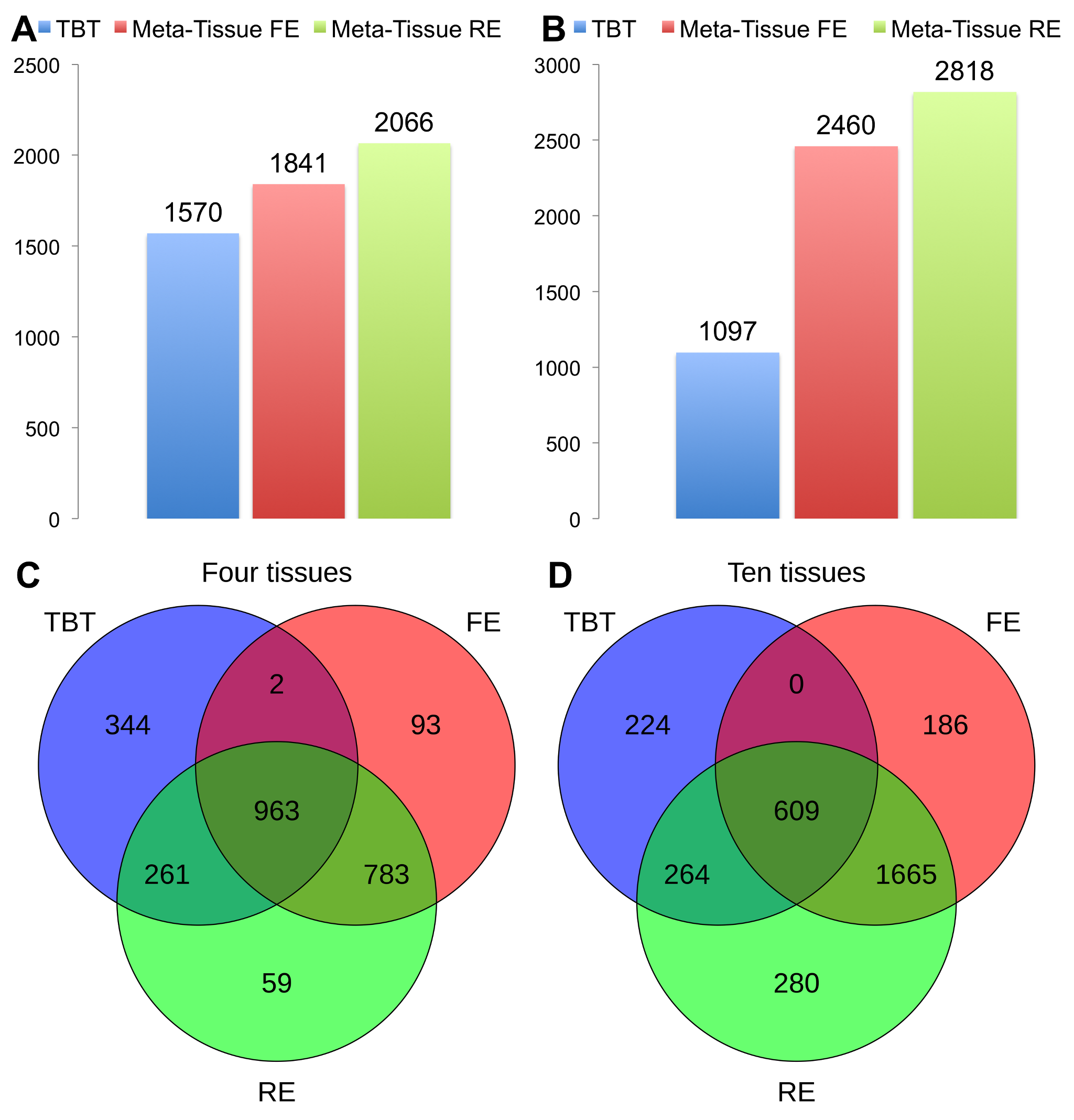

Supplement: Figure S2 — The number of eQTLs detected by the tissue-by-tissue approach (TBT), Meta-Tissue FE, and Meta-Tissue RE in A) four and B) ten tissues of mouse using FDR of 5%, and the overlap of eQTLs detected by the three methods in C) four and D) ten tissues. We consider only cis-eQTLs for the FDR approach, and a pair of SNP-probe for gene expression are considered cis if a SNP and a probe are on the same chromosome. (TIF) [file pgen.1003491.s002.tif]

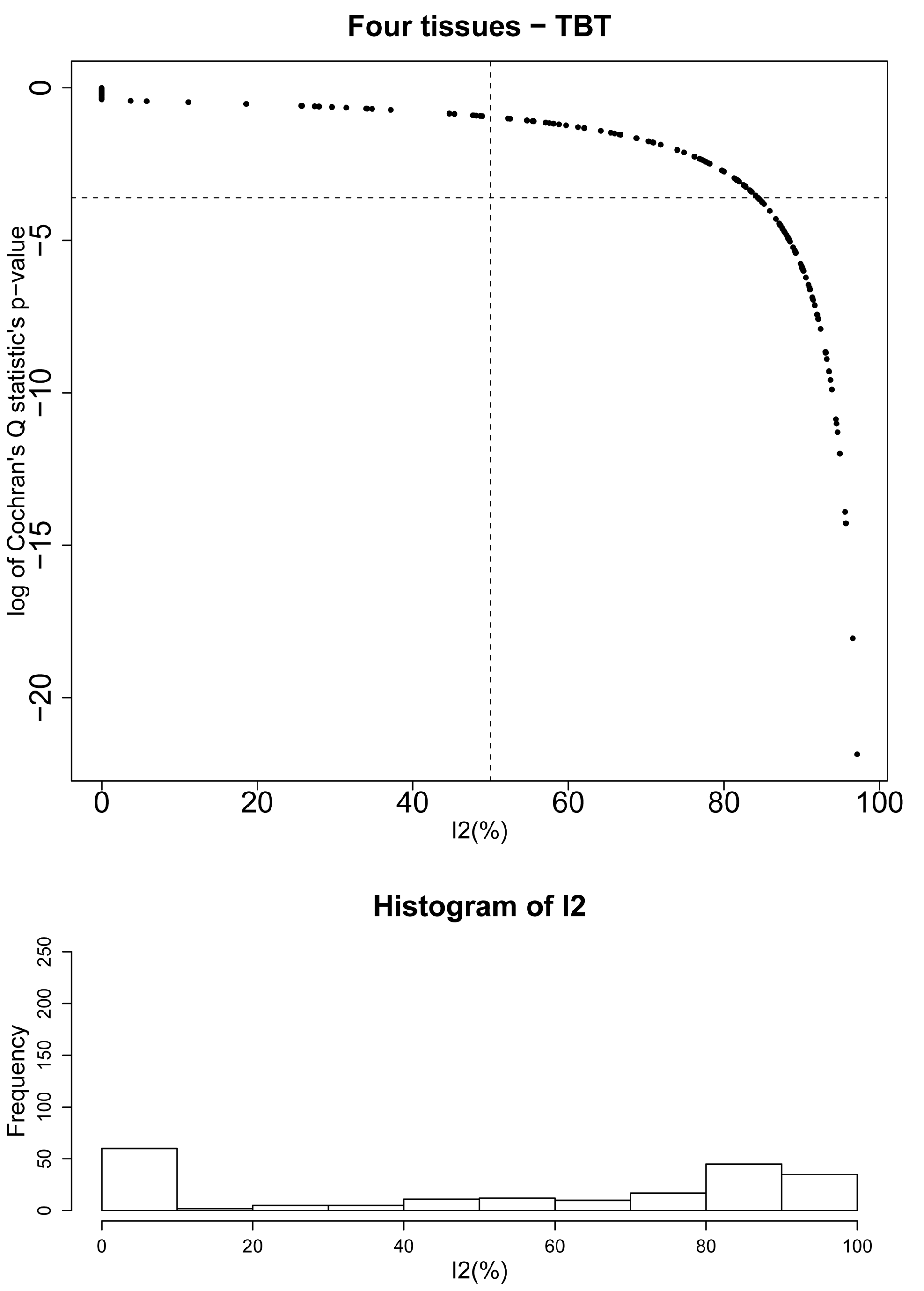

Supplement: Figure S3 — A plot showing heterogeneity of eQTLs detected by the tissue-by-tissue approach. X-axis of the top plot indicates I2 statistic and Y-axis indicates log of p-value of Cochrans Q statistic. The vertical dashed line is drawn at I2 = 50%, and the horizontal dash line is drawn at p-value = 0.05/the number of eQTLs detected. The bottom histogram shows the distribution of I2 statistic. (TIF) [file pgen.1003491.s003.tif]

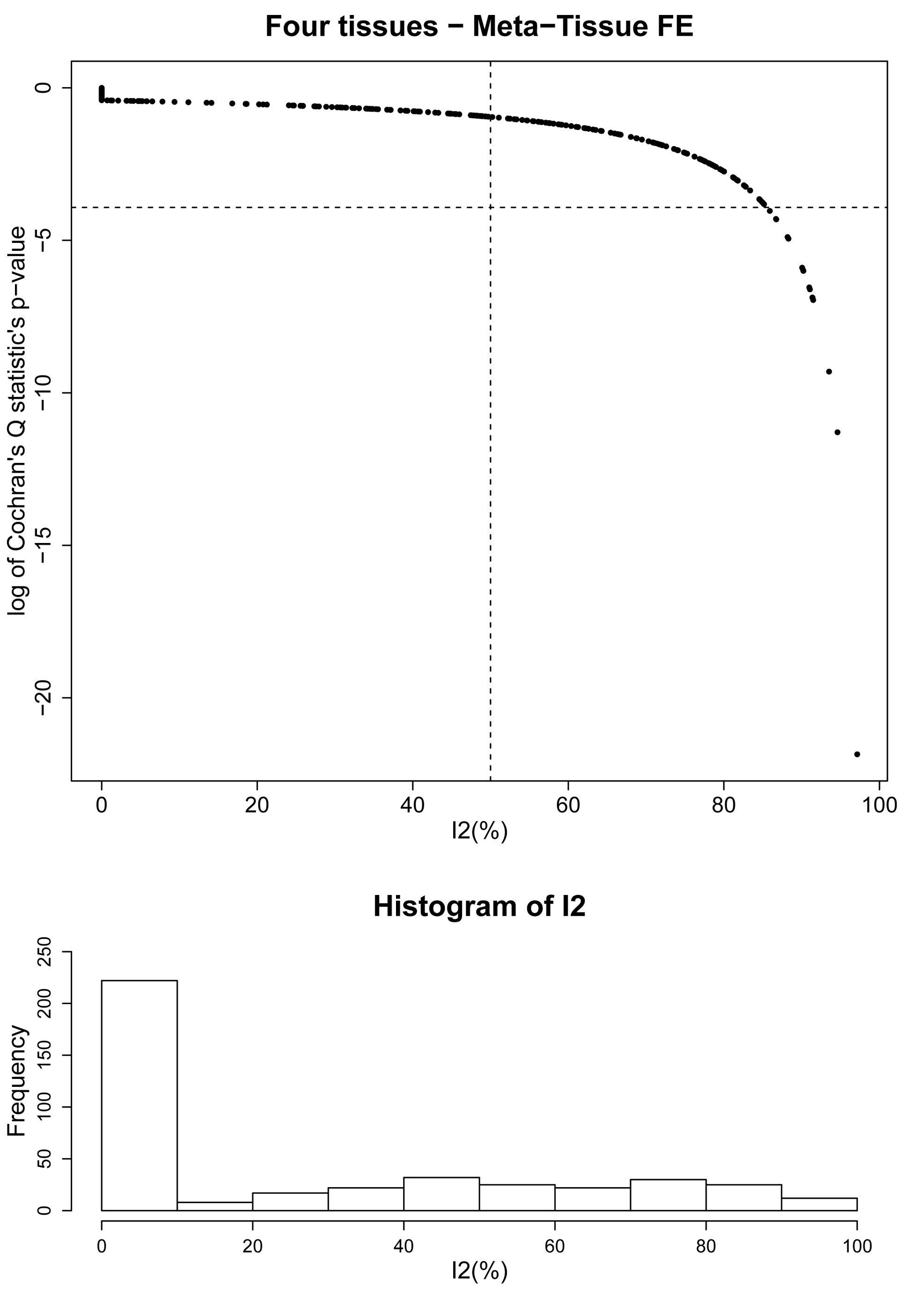

Supplement: Figure S4 — A plot showing heterogeneity of eQTLs detected by Meta-Tissue FE. X-axis of the top plot indicates I2 statistic and Y-axis indicates log of p-value of Cochrans Q statistic. The vertical dashed line is drawn at I2 = 50%, and the horizontal dash line is drawn at p-value = 0.05/the number of eQTLs detected. The bottom histogram shows the distribution of I2 statistic. (TIF) [file pgen.1003491.s004.tif]

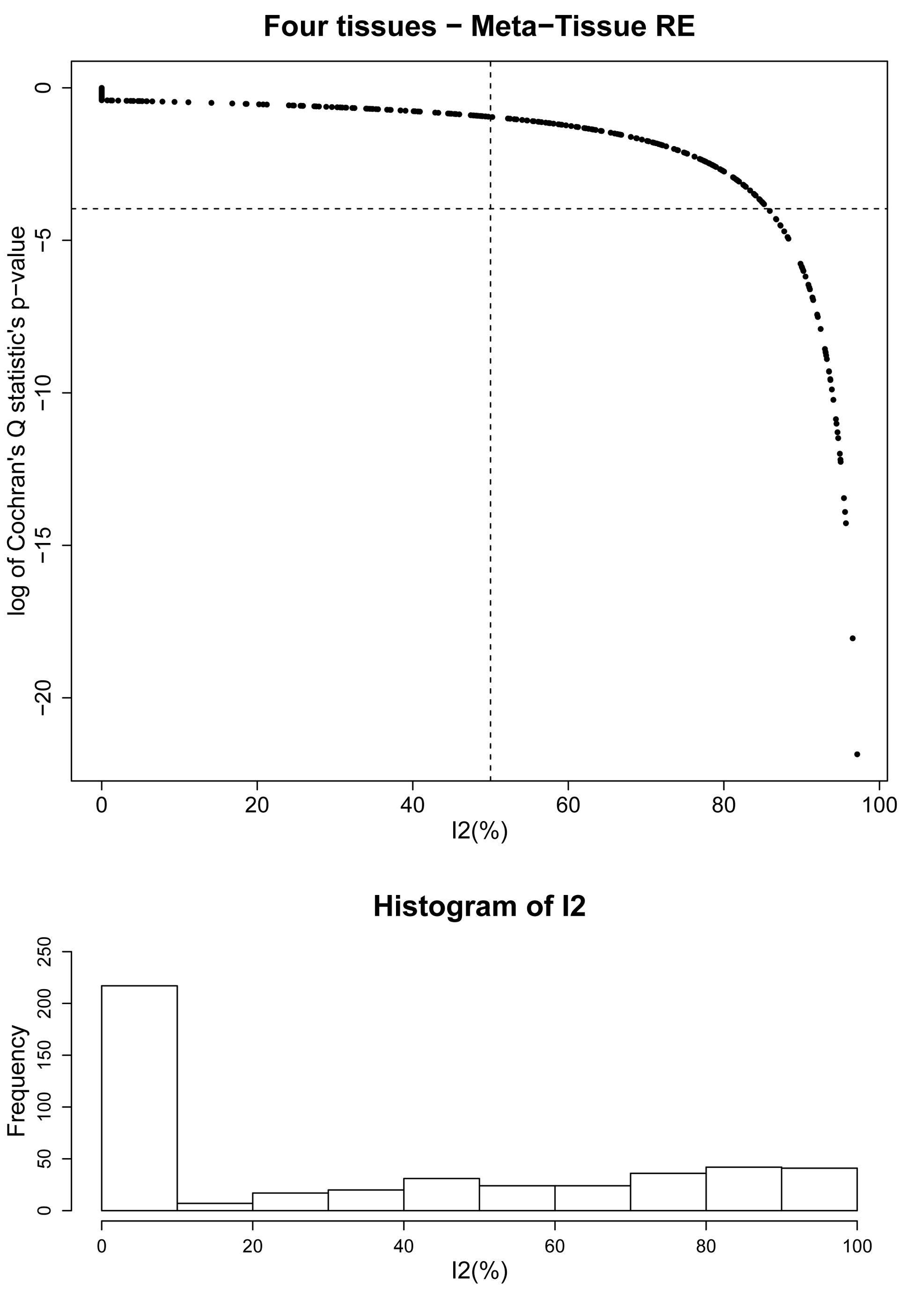

Supplement: Figure S5 — A plot showing heterogeneity of eQTLs detected by Meta-Tissue RE. X-axis of the top plot indicates I2 statistic and Y-axis indicates log of p-value of Cochrans Q statistic. The vertical dashed line is drawn at I2 = 50%, and the horizontal dash line is drawn at p-value = 0.05/the number of eQTLs detected. The bottom histogram shows the distribution of I2 statistic. (TIF) [file pgen.1003491.s005.tif]

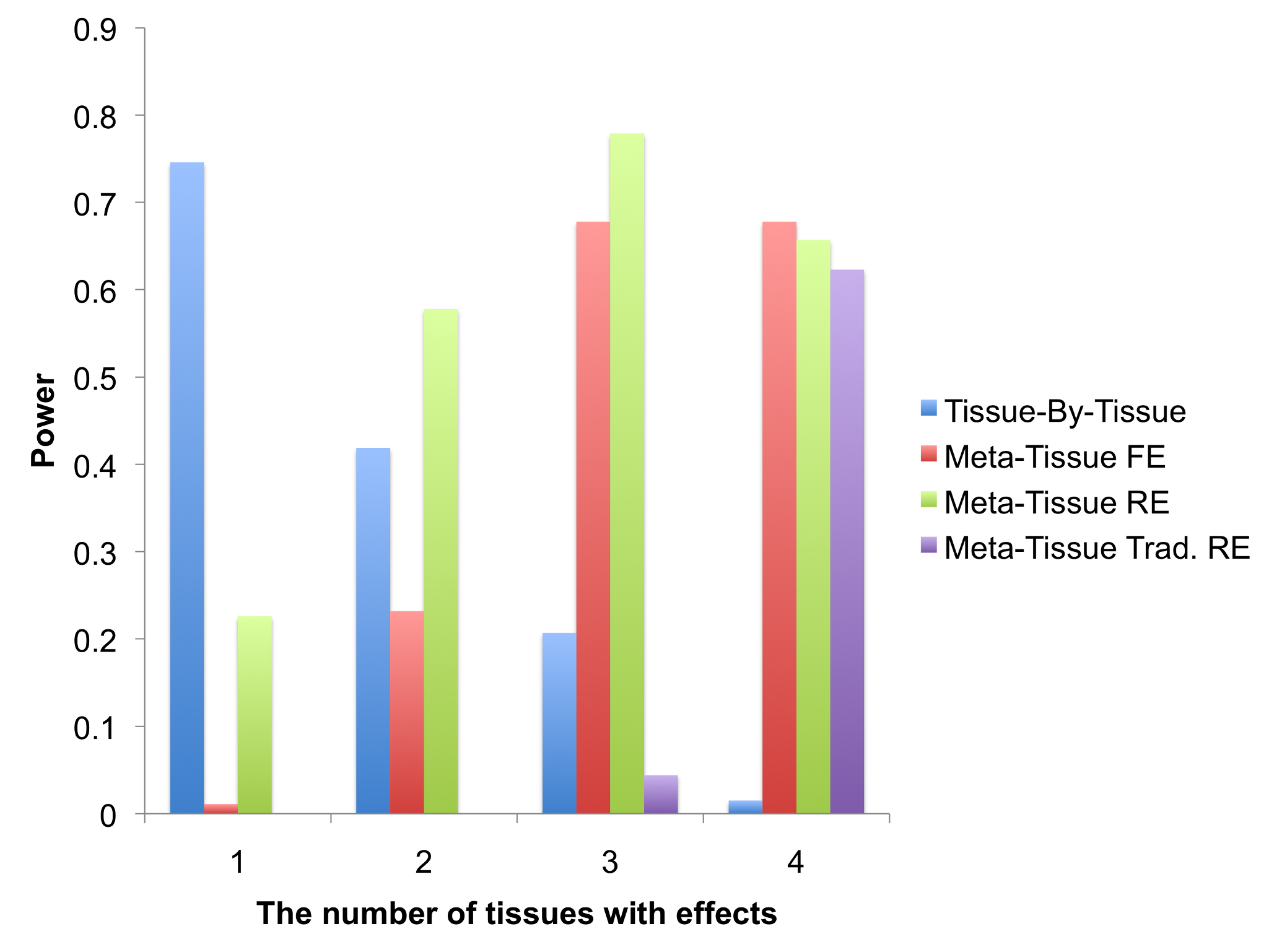

Supplement: Figure S6 — Power comparison between the tissue-by-tissue approach, Meta-Tissue fixed effects model (FE), Meta-Tissue random effects model (RE), and Meta-Tissue traditional random effects model using simulated data. X-axis indicates the number of tissues having effects out of four tissues, and Y-axis is the power. (TIF) [file pgen.1003491.s006.tif]

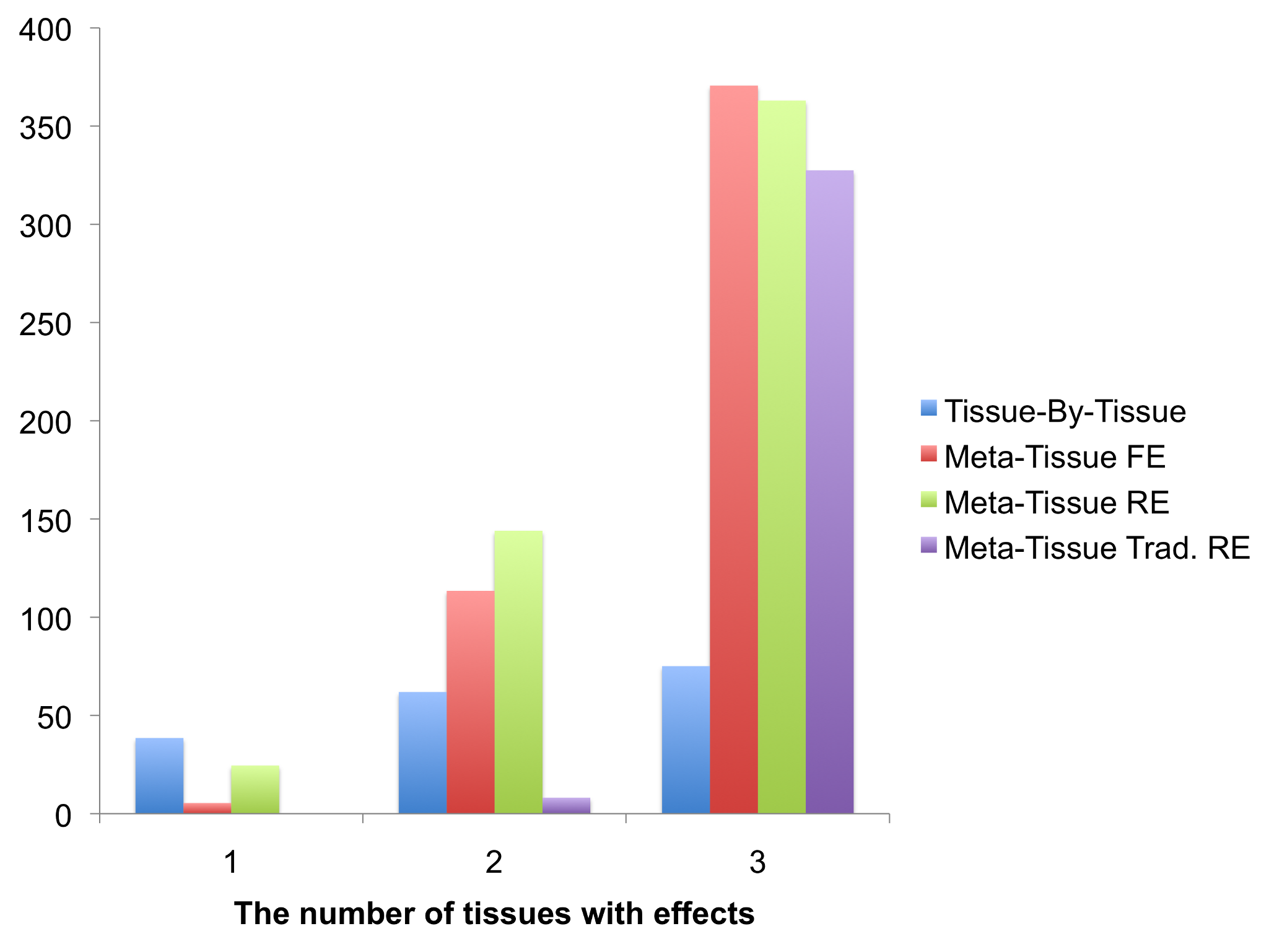

Supplement: Figure S7 — The average number of eQTLs that the tissue-by-tissue approach, Meta-Tissue FE, Meta-Tissue RE, and Meta-Tissue traditional RE recover from three tissues generated from the liver tissue. Effects of eQTLs exist in only two tissues. The original liver tissue has 389 eQTLs. (TIF) [file pgen.1003491.s007.tif]

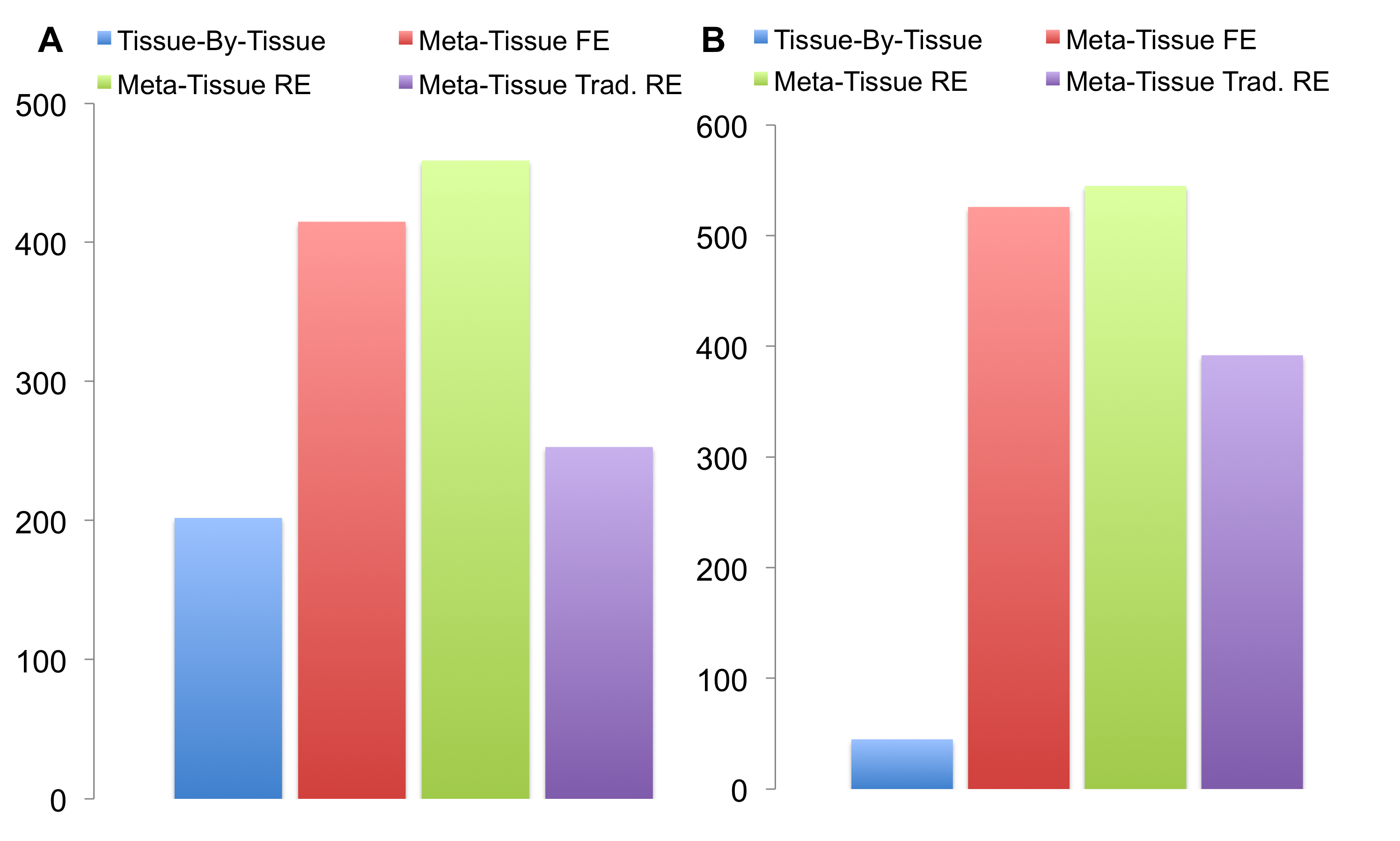

Supplement: Figure S8 — The number of eQTLs detected by the tissue-by-tissue approach, Meta-Tissue FE, Meta-Tissue RE, and Meta-Tissue traditional RE in A) four tissues and in B) ten tissues of mouse. (TIF) [file pgen.1003491.s008.tif]
