# Supplementary material for: Effectively Identifying eQTLs from Multiple Tissues by Combining Mixed Model and Meta-analytic Approaches
Source: PLoS Genet. 2013 Jun 13;9(6):e1003491. doi: 10.1371/journal.pgen.1003491 (PMC3681686; doi:10.1371/journal.pgen.1003491)
Supplement: Text S1 — False positive rates of Meta-Tissue. (PDF) [file pgen.1003491.s010.pdf]

Effectively identifying eQTLs from multiple tissues by combining mixed model and meta-analytic approaches Supporting Information Text S1

Jae Hoon Sul[1,6], Buhm Han[2,3,6], Chun Ye[3,6], Ted Choi[4], Eleazar Eskin[1,5,*]

1 Computer Science Department, University of California, Los Angeles, California, USA

2 Division of Genetics, Brigham & Women's Hospital, Harvard Medical School, Boston, MA, USA

3 Broad Institute of MIT and Harvard, Cambridge, Massachusetts, USA

4 Predictive Biology, Inc., San Diego, California, USA

5 Department of Human Genetics, University of California, Los Angeles, California , USA

6 These authors contributed equally to this work

∗ **E-mail: eeskin@cs.ucla.edu**

False positive rates of meta analysis

To measure the false positive rate of our proposed method, we simulate a multiple tissue dataset where there is no eQTL; a SNP has no effect. We consider 10,000 gene expression levels and 100 SNPs simulating a million pairs of gene and SNP. The number of tissues is four, and we use SNP data from real four tissue dataset from mouse where we have 50 individuals per each tissue. In this dataset, 34% of individuals are shared between two tissues on average, as discussed in the Results section. To generate gene expression for individuals, we use Equation (1) where $\sigma_v = \sigma_e = 0.5$. $\mathbf{D}$ matrix is the same as $\mathbf{D}$ matrix used in the four tissues analysis. We use `mvtnorm` package in `R` to generate gene expression from the multivariate normal distribution.

Table 1 shows that Meta-Tissue that uses the linear mixed model to account for the correlation in gene expression between tissues has correct false positive rates. We use the significance threshold of 0.05, and Meta-Tissue FE and RE have false positive rates of 0.533 and 0.0417, respectively. We also measure the false positive rate when meta-analysis methods do not use the linear mixed model, but the linear model that assumes all tissues are independent. Table 1 shows that meta-analysis methods have inflation of false positives in this case; Meta-Tissue FE and RE have false positive rates of 0.11 and 0.097. This shows that meta-analysis methods need to consider that individuals are shared across the tissues when

combining results from multiple tissues.

Methods	Linear mixed model	Linear model
Meta-Tissue FE	0.053323	0.10992
Meta-Tissue RE	0.041781	0.0972

Table 1. False positive rates of Meta-Tissue FE and RE using the linear mixed model and the linear model.
