# Supplementary material for: Effectively Identifying eQTLs from Multiple Tissues by Combining Mixed Model and Meta-analytic Approaches
Source: PLoS Genet. 2013 Jun 13;9(6):e1003491. doi: 10.1371/journal.pgen.1003491 (PMC3681686; doi:10.1371/journal.pgen.1003491)
Supplement: Text S2 — Practical issues in combining mixed model and meta-analysis. (PDF) [file pgen.1003491.s011.pdf]

Effectively identifying eQTLs from multiple tissues by combining mixed model and meta-analytic approaches Supporting Information Text S2

Jae Hoon Sul[1,6], Buhm Han[2,3,6], Chun Ye[3,6], Ted Choi[4], Eleazar Eskin[1,5,*]

1 Computer Science Department, University of California, Los Angeles, California, USA

2 Division of Genetics, Brigham & Women's Hospital, Harvard Medical School, Boston, MA, USA

3 Broad Institute of MIT and Harvard, Cambridge, Massachusetts, USA

4 Predictive Biology, Inc., San Diego, California, USA

5 Department of Human Genetics, University of California, Los Angeles, California , USA

6 These authors contributed equally to this work

∗ **E-mail: eeskin@cs.ucla.edu**

Practical issues in combining mixed model and meta-analysis

t-distributed effect size estimates

There are subtle issues in our framework combining mixed model and meta-analysis. First, the effect size estimates from linear model or mixed model are typically t-distributed, while most of meta-analysis methods assume normally distributed effect sizes. Let $\hat{\beta}$ and $\mathrm{var}(\hat{\beta})$ be the effect size estimate and the variance estimate from a linear model. Assume that under the null, $\frac{\hat{\beta}}{\sqrt{\mathrm{var}(\hat{\beta})}}$ will approximately follow t-distribution with k degree of freedom. The p-value is calculated

$$p_t = 2\left(1 - \Phi_{t(k)}\left(\frac{|\hat{\beta}|}{\sqrt{\mathrm{var}(\hat{\beta})}}\right)\right)$$

where $\Phi_{t(k)}$ is the cummulative density function of the t-distribution with k degree of freedom. If we directly use $\hat{\beta}$ and $\mathrm{var}(\hat{\beta})$ in the meta-analysis approach assuming normally distributed effect size, false positive rate will increase. This issue is particularly important in model organisms where the sample size is moderate.

To correct for this, we use simple heuristic replacing $\sqrt{\text{var}(\hat{\beta})}$ with

$$\frac{|\hat{\beta}|}{|\Phi^{-1}(p_t/2)|}$$

where Φ^{-1} is the inverse of the cummulative density function of the standard normal distribution. That is, we increase the variance of $\hat{\beta}$ according to the difference between the t-distribution and the normal distribution to prevent an excessive false positive rate in the meta-analysis.

Differences in error models

Another issue is that our approach simultaneously considers all tissues using Equation (3), but the error model is slightly different from the tissue-by-tissue approach in Equation (2). In the tissue-by-tissue approach, the error $\mathbf{e} \sim N(0, \sigma^2\mathbf{I})$ is fit in each tissue separately, while in our new approach, the error is fit in all tissues together. Certainly, the tissue-by-tissue model is more desirable because we cannot always expect that the true variance of error term (σ^2) to be the same across tissues. In other words, in our new framework, we are imposing an unrealistic assumption that the error variance is the same for all tissues, or constant error variance assumption (CEVA). We find that our approach is often less powerful when the truth deviates from CEVA. To compensate for the effect of this assumption, we apply the following idea. Before using our mixed model in Equation (3), we standardize the gene expressions in each tissue to follow $\mathcal{N}(0, 1)$. Note that this does not completely solve the problem because gene expression values include not only the error term but also the genetic effects.

To further correct for the effect of our assumption, we use the following heuristic. We first run tissue-by-tissue approach to obtain the effect size estimate $\hat{\beta}_{TBT}$ and its standard error STD_{TBT}. Second, we run our mixed model in Equation (3) assuming that $\sigma_v^2 = 0$. That is, we intentionally ignore the correlations of multiple tissue expressions from the same individuals. Under this simplified model, the estimate $\hat{\beta}_{COMB}$ turns out to be exactly the same as $\hat{\beta}_{TBT}$. Let STD_{COMB} be the standard error of $\hat{\beta}_{COMB}$ under this model. Although the effect size estimates are the same ($\hat{\beta}_{TBT} = \hat{\beta}_{COMB}$), their standard errors are different in two models because their error models are different. Therefore, the ratio between the two standard errors can be a measure of the effect of CEVA.

Finally, we run our standard mixed model by estimating σ_v^2 and σ_e^2 using the EMMA package. Let $\hat{\beta}_{MIX}$ and STD_{MIX} be the effect size estimate and its standard error under this model. Then we heuris-

tically obtain a new standard error

$$STD_{NEW} = STD_{MIX} \cdot \frac{STD_{TBT}}{STD_{COMB}}$$

That is, we correct for the effect of CEVA using the ratio between STD_{TBT} and STD_{COMB}. What we use in the subsequent meta-analysis are $\hat{\beta}_{MIX}$ and STD_{NEW}. We find that this simple heuristic effectively corrects for the effect of CEVA in many cases.
